# The ZuCo benchmark on cross-subject reading task classification with EEG and eye-tracking data

**DOI:** 10.3389/fpsyg.2022.1028824

**Published:** 2023-01-12

**Authors:** Nora Hollenstein, Marius Tröndle, Martyna Plomecka, Samuel Kiegeland, Yilmazcan Özyurt, Lena A. Jäger, Nicolas Langer

**Affiliations:** ^1^Center for Language Technology, University of Copenhagen, Copenhagen, Denmark; ^2^Department of Psychology, University of Zurich, Zurich, Switzerland; ^3^Department of Computer Science, ETH Zurich, Zurich, Switzerland; ^4^Department of Computational Linguistics, University of Zurich, Zurich, Switzerland; ^5^Department of Computer Science, University of Potsdam, Potsdam, Germany

**Keywords:** reading task classification, eye-tracking, EEG, machine learning, reading research, cross-subject evaluation

## Abstract

We present a new machine learning benchmark for reading task classification with the goal of advancing EEG and eye-tracking research at the intersection between computational language processing and cognitive neuroscience. The benchmark task consists of a cross-subject classification to distinguish between two reading paradigms: normal reading and task-specific reading. The data for the benchmark is based on the Zurich Cognitive Language Processing Corpus (ZuCo 2.0), which provides simultaneous eye-tracking and EEG signals from natural reading of English sentences. The training dataset is publicly available, and we present a newly recorded hidden testset. We provide multiple solid baseline methods for this task and discuss future improvements. We release our code and provide an easy-to-use interface to evaluate new approaches with an accompanying public leaderboard: www.zuco-benchmark.com.

## 1. Introduction

Reading plays a fundamental role in the acquisition of information (e.g., encyclopedias) and communication (e.g., emails). As we read, our eyes gaze through the written sentences in a sequence of fixations and high-velocity saccades to extract visual information which are forwarded to the brain to obtain meaning. Thus, assessing where a person looks during reading while recording brain activity non-invasively with electroencephalography (EEG) provides powerful behavioral and physiological measures for cognitive neuroscience to further the understanding of human language processing. Most previous experimental reading research has used hand-picked reading materials in highly controlled experimental settings (Brennan, 2016; Nastase et al., 2020). The neural correlates of reading have traditionally been studied with serial word-by-word presentation with a fixed presentation time, which eliminates important aspects of the natural reading process and precludes direct comparisons between neural activity and oculomotor behavior (Dimigen et al., 2011; Kliegl et al., 2012). The electrical neural correlates of normal reading of naturally occurring real-world sentences have been investigated less frequently due to a number of methodological challenges related to identifying the exact timing and type of visual stimuli presented during reading.

Because of recent methodological progress in stimulus presentation and data preprocessing (Dimigen et al., 2011; Ehinger and Dimigen, 2019), an excellent temporal resolution, and low costs, co-registered EEG, and eye-tracking have become important tools for studying the temporal dynamics of naturalistic reading (Frey et al., 2018; Hollenstein et al., 2018). Fixation-related potentials (FRPs), the evoked electrical responses time-locked to the onset of fixations, have become important tools for researchers to study various topics including free-viewing visual perception (e.g., Rämä and Baccino, 2010), brain-computer interfaces (e.g., Finke et al., 2016), and natural reading (e.g., Degno et al., 2019). In naturalistic reading paradigms, FRPs allow the study of the neural dynamics of how new information from a currently fixated word affects the ongoing language comprehension process.

In this work, we leverage these novel methodological advances to offer a machine learning (ML) benchmark challenge, formulated as a cross-subject classification task, to identify two reading tasks as accurately as possible. Specifically, the challenge is to discriminate between normal reading (with the only task of reading comprehension) and task-specific reading (TSR; with the purpose of finding specific information in the text) from eye-tracking and EEG data. Decoding mental states and detecting specific cognitive processes occurring in the brain during different reading tasks (i.e., *reading task classification*) are important challenges in cognitive neuroscience as well as in natural language processing (NLP). Applications of reading task classification include measuring attention and engagement (Miller, 2015; Abdelrahman et al., 2019), detecting proper reading vs. skimming (Biedert et al., 2012), as well as applications related to intent recognition within brain computer interfaces (Schalk et al., 2008). Other studies have demonstrated that recognizing reading patterns for estimating reading effort can improve the diagnosis of reading impairments such as dyslexia (Rello and Ballesteros, 2015; Raatikainen et al., 2021) and attention deficit disorder (Tor et al., 2021). Furthermore, it has been shown that using EEG and eye-tracking signals facilitates the prediction workload (Lobo et al., 2016) and investigation of language learning (Notaro and Diamond, 2018).

The accurate distinction of the cognitive processes occurring in different reading tasks is also important for ML and NLP. Identifying specific reading patterns can improve models of human reading and provide insights into human language understanding and how we perform linguistic tasks. This knowledge can then be applied to ML algorithms for NLP (e.g., information extraction applications). Computational models of language understanding can be adapted based on the insights from different reading and language processing tasks. Therefore, the identification of reading intents can be beneficial for computational methods of language understanding, but also for applications such as digital assistant tools, e.g., supporting translation processes, understanding how learners approach tasks in adaptive e-learning, and inferring document relevance.

A crucial potential of human physiological data in the context of NLP is that it can be leveraged to understand and to improve the manual labeling process required for generating training samples for supervised ML. For instance, Tokunaga et al. (2017) analyze eye-tracking data during the annotation of text to find effective gaze features for a specific NLP task and Tomanek et al. (2010) build cost models for active learning scenarios based on insights from eye-tracking data.

Reading task classification can help to improve the labeling processes by detecting tiredness from brain activity data and eye-tracking data, and subsequently to suggest breaks or task switching, or by using cognitive data directly to (pre-)annotate samples used for training ML models. If we can find and extract the relevant aspects of text understanding and annotation directly from the source, i.e., eye-tracking and brain activity signals during reading, we can potentially replace this expensive manual labeling work with ML models trained on physiological activity data recorded from humans while reading. Therefore, successful reading task classification could support the reduction of manual labor, improving label quality in ML systems as well as the job quality of annotators.

Essential for using neurophysiological signals to advance NLP is the availability of a large dataset providing concurrent measures of eye-tracking and EEG data, as well as ground truth labels for ML tasks. For the present benchmark, this is possible by leveraging a naturalistic dataset of reading English sentences, the Zurich Cognitive Language Processing Corpus (Hollenstein et al., 2018, 2020). The ZuCo dataset is publicly available and has recently been used in a variety of applications including leveraging EEG and eye-tracking data to improve NLP tasks (Barrett et al., 2018; Mathias et al., 2020; McGuire and Tomuro, 2021), evaluating the cognitive plausibility of computational language models (Hollenstein et al., 2019b; Hollenstein and Beinborn, 2021), investigating the neural dynamics of reading (Pfeiffer et al., 2020), developing models of human reading (Bautista and Naval, 2020; Bestgen, 2021).

Recently, ZuCo has also been leveraged for an ML competition on eye-tracking prediction (Hollenstein et al., 2021a). This competition revolves around a different task with a focus on computational language models in the field of natural language processing. The goal was to predict word-level eye-tracking features from normal reading such as mean fixation duration and fixation probability in a regression task. This shows that the ZuCo dataset has been used successfully for a wide range of ML tasks.

Moreover, the results of previous single-subject models for reading task classification (Hollenstein et al., 2021c; Mathur et al., 2021) emphasize the potential of this task, but also highlight the performance gap between research-oriented single-subject models and more realistic cross-subject scenarios. The proposed benchmark therefore addresses this gap by focusing on the latter to improve the inter-subject generalization capabilities of these machine learning models. The recording of a new hidden testset with additional participants enables us to test this task in a suitable manner. Furthermore, by applying state-of-the-art EEG recording and preprocessing techniques, we ensure that this benchmark relies on a strong foundation, so that the resources and efforts of the research community can be spent wisely.

To conclude, the contributions of our work can be summarized as follows: First, we formulate a benchmark task for applying ML techniques to an important problem in cognitive science, namely, the classification of cognitive tasks. Second, we provide the data^1^ and code^2^ to reproduce our experiments. We provide a public benchmark and leaderboard on a new held-out test data. All information can be found here: www.zuco-benchmark.com. Finally, we propose and discuss models using various feature sets as baseline models for this benchmark task. We present detailed analyses of the results for both eye-tracking and EEG features and discuss the model performances.

## 2. Methods

The basis for this ML benchmark task is the Zurich Cognitive Language Processing Corpus 2.0 (ZuCo 2.0). ZuCo 2.0 was originally published in Hollenstein et al. (2020). In short, this corpus contains gaze and brain activity data of 18 participants reading 739 English sentences, 349 in a normal reading paradigm, and 390 in a task-specific paradigm, in which the participants actively search for a semantic relation type in the given sentence as a linguistic annotation task. This new dataset provides experiments designed to analyze the differences in cognitive processing between normal reading and task-specific reading.

In previous work, we recorded a first dataset (i.e., ZuCo 1.0) of simultaneous eye-tracking and EEG during natural reading (Hollenstein et al., 2018). ZuCo 1.0^3^ consists of three reading tasks, two of which contain very similar reading material and experiments as presented in the current work. However, for ZuCo 1.0 the normal reading and task-specific reading paradigms were recorded in different sessions on different days. Therefore, the recorded data from ZuCo 1.0 is not appropriate as a means of comparison between normal reading and task-specific reading, since the differences in the brain activity data might result mostly from the different sessions due to the sensitivity of EEG. Therefore, while the data is available in the same format, it is not recommended to be used for this benchmark task. In the following section, we describe the compilation of the ZuCo 2.0 dataset.

### 2.1. Reading materials

During the recording session, the participants read a total of 739 sentences that were selected from the Wikipedia corpus provided by (Culotta et al., 2006). This corpus was chosen because it provides annotations of semantic relations. Relation detection is a high-level semantic language understanding task requiring complex cognitive processing. ZuCo 2.0 includes seven of the originally defined relation types: *political_affiliation, education, founder, wife/husband, job_title, nationality*, and *employer*. The sentences were chosen with similar sentence lengths and Flesch reading ease scores (Flesch, 1948) between the two reading tasks. The Flesch score indicates how difficult an English text passage is to understand based on its structural characteristics, i.e., number of words and number of syllables. A higher Flesch score means the text is easier to read. The dataset statistics are shown in Table 1.

**Table 1:** Descriptive statistics of reading materials (SD, standard deviation), including Flesch readibility scores.

	**NR**	**TSR**
Sentences	349	390
Sent. length	Mean (SD), range	Mean (SD), range
	19.6 (8.8), 5–53	21.3 (9.5), 5–53
Total words	6,828	8,310
Word types	2,412	2,437
Word length	Mean (SD), range	Mean (SD), range
	4.9 (2.7), 1–29	4.9 (2.7), 1–21
Flesch score	55.38	50.76

**Figure 1:**
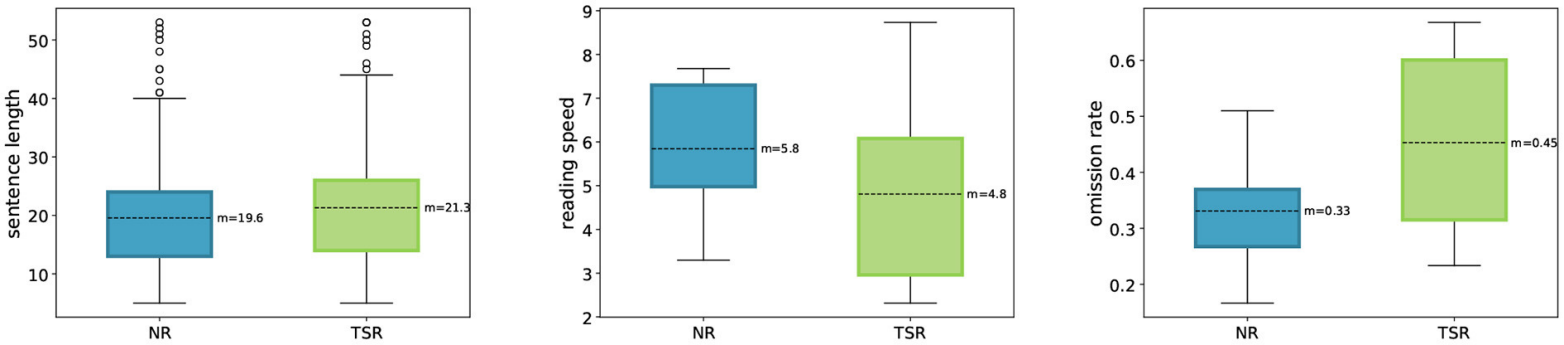
Sentence length (words per sentence), reading speed (seconds per sentence) and omission rate (percentage of words not fixated) comparison between normal reading (NR) and task-specific reading (TSR) of the sentence in ZuCo 2.0.

Of the 739 sentences, the participants read 349 sentences in a normal reading paradigm and 390 sentences in a task-specific reading paradigm, in which they had to determine whether a certain relation type occurred in the sentence or not. Table 2 shows the distribution of the different relation types in the sentences of the task-specific annotation paradigm. Purposefully, there are 63 duplicates between the normal reading and the task-specific sentences (8% of all sentences). The intention of these duplicate sentences is to provide a set of sentences read twice by all participants with a different task in mind. Hence, this enables the comparison of eye-tracking and brain activity data when reading normally and when annotating specific relations. During both tasks, the participants were able to read in their own speed, using a control pad to move to the next sentence and to answer the control questions, which allowed for natural reading. Since all subject read at their own personal pace, the reading speed varies between subjects. Figure 1 shows the average sentence length, reading speed, and omission rate for each task.The sentence length (i.e., the number of words per sentence) was controlled in the selection of reading materials, so that it would not differ significantly between the two tasks (NR mean = 19.6, SD = 8.8; TSR mean = 21.3, SD = 9.5; *p* = 0.02 in a two-sided *t*-test).

**Table 2:** Distribution of relation types in the task-specific reading.

**Relation type**	**Sentences**
Political affiliation	45 (9)
Education	72 (10)
Wife	54 (12)
Job title	65 (11)
Employer	54 (10)
Nationality	60 (8)
Founder	40 (8)
**Total**	**390 (68)**

The right column contains the number of sentences, and the number control sentences without a relation in brackets.

#### 2.1.1. Normal reading

In the first task, participants were instructed to read the sentences naturally, without any specific task other than comprehension. An example sentence is “He served in the United Stated Army in World War II, then got a law degree from Tulane University.” The control condition for this task consisted of single-choice questions about the content of the previous sentence. Twelve percent of randomly selected sentences were followed by a comprehension question with three answer options on a new screen, for example, “Which university did he get his degree from? (1) Austin University, (2) Tulane University, (3) Louisiana State University.”

#### 2.1.2. Task-specific reading

In the second task, the participants were instructed to search for a specific semantic relation in each sentence they read. Instead of comprehension questions, the participants had to decide for each sentence whether it contains the relation or not, i.e., they were actively annotating each sentence. An example sentence containing the relation *founder* is “After this initial success, Ford left Edison Illuminating and, with other investors, formed the Detroit Automobile Company.” Seventeen percent of the sentences did not include the particular relation type and were used as control conditions. All sentences within one recording block involved the same relation type. Each block was preceded by a short practice round, which described the relation type and was followed by three sample sentences, so that the participants would be familiar with the respective relation type.

### 2.2. Linguistic assessment

As a linguistic assessment, the vocabulary and language proficiency of the participants was tested with the LexTALE test (Lexical Test for Advanced Learners of English, Lemhöfer and Broersma, 2012). This is an unspeeded lexical decision task designed for intermediate to highly proficient language users. The average LexTALE score over all participants was 88.54%. Moreover, we also report the scores the participants achieved with their answers to the reading comprehension control questions and their relation annotations. The detailed scores for all participants are also presented in Table 3.

**Table 3:** Subject demographics for ZuCo 2.0, LexTALE scores, scores of the comprehension questions, and individual reading speed (i.e., seconds per sentence) for each task.

**ID**	**Age**	**Gender**	**LexTALE**	**Comp. scores**		**Reading speed**	
				**NR**	**TSR**	**NR**	**TSR**
YAC	32	female	76.25%	82.61%	83.85%	5.27	4.96
YAG	47	female	93.75%	91.30%	56.92%	7.64	8.73
YAK	31	female	100.00%	74.07%	96.41%	3.83	5.89
YDG	51	male	100.00%	91.30%	96.67%	4.97	3.93
YDR	25	male	85.00%	78.26%	96.92%	4.32	2.32
YFR	27	male	85.00%	89.13%	94.36%	6.48	4.79
YFS	39	male	90.00%	91.30%	96.15%	3.96	2.85
YHS	31	male	90.00%	78.26%	97.69%	3.30	2.40
YIS	52	male	97.50%	89.13%	98.46%	5.82	2.58
YLS	34	female	93.75%	91.30%	92.31%	5.57	5.85
YMD	31	female	100.00%	86.96%	95.64%	7.50	6.24
YRK	29	female	85.00%	97.83%	96.15%	7.35	7.70
YRP	23	female	82.50%	78.26%	90.00%	7.14	8.37
YSD	34	male	95.00%	93.48%	94.36%	5.01	2.87
YSL	32	female	71.25%	84.78%	83.85%	6.73	6.14
YTL*	36	male	81.25%	80.43%	94.10%	7.48	3.23
**Mean**	**34**	**44% m**.	**88.54%**	**86.36%**	**91.94%**	**5.84**	**4.81**

The * next to the subject ID marks a bilingual subject.

### 2.3. Participants

The subjects from ZuCo 2.0 are provided as training data for the current benchmark. For the ZuCo 2.0, we recorded data from 19 participants and discarded the data of one of them due to technical difficulties with the eye-tracking calibration. Another two subjects were discarded during data cleaning and preprocessing. Thus, we share the data of these 16 participants. All participants are healthy adults (between 23 and 52 years old; 10 females). Details on subject demographics can be found in Table 3. Their native language is English, originating from Australia, Canada, UK, USA or South Africa. Two participants are left-handed and three participants wear glasses for reading. All participants gave written consent for their participation and the re-use of the data prior to the start of the experiments. The study was conducted under approval by the Ethics Commission of the University of Zurich.

#### 2.3.1. ZuCo 2.0 held-out testset

To provide a true hidden dataset for the current benchmark, we recorded data from 10 additional participants (i.e., a held-out testset). They underwent the identical procedure as in the ZuCo 2.0 dataset. All participants are healthy adults [mean age = 31.8 (SD = 5.11), four females]. All participants are right-handed. Their native language is English, originating from UK, Canada or USA. For an overview on subjects demographics, comprehension scores and reading speed please refer to Table 4. All participants gave written consent for their participation and the re-use of the data prior to the start of the experiments.

**Table 4:** Subject demographics for the new held-out test dataset, LexTALE scores, scores of the comprehension questions, and individual reading speed (i.e.,seconds per sentence) for each task.

**ID**	**Age**	**Gender**	**LexTALE**	**Comp. scores**		**Reading speed**	
				**NR**	**TSR**	**NR**	**TSR**
XAH	25	female	95.25%	91.30%	93.58%	5.58	3.94
XBB	37	male	95.75%	82.60%	93.84%	6.88	5.67
XBD	32	male	89.00%	91.30%	96.15%	7.31	4.48
XDT	25	male	97.50%	86.95%	93.85%	8.24	8.54
XLS	28	male	85.00%	89.13%	94.87%	7.52	5.68
XPB	29	male	97.50%	86.95%	91.02%	7.87	6.53
XSE	31	female	90.00%	89.13%	96.15%	7.23	3.75
XSS	42	female	97.50%	89.13%	96.67%	7.49	6.21
XTR	34	female	93.75%	89.13%	96.15%	9.18	5.91
XWS	35	male	100.00%	89.13%	95.64%	6.65	4.29
**Mean**	**31.8**	**60% m**.	**94.13%**	**88.48%**	**94.79%**	**7.40**	**5.50**

### 2.4. Procedure

Data acquisition took place in a sound-attenuated and dark experiment room. Participants were seated at a distance of 68 cm from a 24-inch monitor (ASUS ROG, Swift PG248Q, display dimensions 531 × 299 mm, resolution 800 × 600 pixels resulting in a display: 400 × 298.9 mm, a vertical refresh rate of 100 Hz). All sentences were presented at the same position on the screen and could span multiple lines. The sentences were presented in black on a light gray background with font size 20-point Arial, resulting in a letter height of 0.8 mm. The experiment was programmed in MATLAB 2016b (MathWorks, Inc. 2000), using PsychToolbox (Brainard, 1997). A stable head position was ensured *via* a chin rest. Participants were instructed to stay as still as possible during the recordings to avoid motor EEG artifacts. Participants completed the tasks sitting alone in the room, while two research assistants were monitoring their progress in the adjoining room. All recording scripts including detailed participant instructions are available alongside the data. During both tasks, the participants were able to read in their own speed, using a control pad to move to the next sentence and to answer the control questions, which allowed for natural reading. All 739 sentences were recorded in a single session for each participant. The duration of the recording sessions was between 100 and 180 min, depending on the time required to set up and calibrate the devices, and the personal reading speed of the participants. Participants were also offered snacks and water during the breaks and were encouraged to rest. We recorded 14 blocks of ~50 sentences, alternating between tasks: 50 sentences of normal reading, followed by 50 sentences of task-specific reading. The order of blocks and sentences within blocks was identical for all subjects. Each sentence block was preceded by a practice round of three sentences and followed by a short break to ensure a clear separation between the reading tasks. For the held-out test dataset, all blocks were merged and the order of the sentences was shuffled before sharing the data on OSF. This is done to prohibit the possibility that challenge participants would simply train a model to identify an experimental block rather than the type of reading for each sentence.

### 2.5. Data acquisition

#### 2.5.1. Eye-tracking acquisition

Eye movements and pupil size were recorded with an infrared video-based eye tracker (EyeLink 1000 Plus, SR Research) at a sampling rate of 500 Hz and an instrumental spatial resolution of 0.01°. The eye tracker was calibrated with a nine-point grid at the beginning of the session and re-validated before each block of sentences. Participants were instructed to keep their gaze on a given point until it disappeared. If the average error of all points (calibration vs. validation) was below 1° of visual angle, the positions were accepted. Otherwise, the calibration was redone until this criterion was reached.

#### 2.5.2. EEG acquisition

We recorded the high-density EEG data at a sampling rate of 500 Hz with a bandpass of 0.1–100 Hz, using a 128-channel EEG Geodesic Hydrocel system (Electrical Geodesics). The *Cz* electrode served as a recording reference. The impedance of each electrode was checked before recording and was kept below 40 *kΩ*. Additionally, electrode impedance levels were checked after every third block of 50 sentences (approximately every 30 min) and reduced if necessary.

### 2.6. Data preprocessing and feature extraction

#### 2.6.1. Eye-tracking preprocessing and feature extraction

##### 2.6.1.1. Eye-tracking preprocessing

The eye tracker computed eye position data and identified events such as saccades, fixations, and blinks. Saccade onsets were detected using the eye-tracking software default settings: acceleration larger than 8,000°/s2, a velocity above 30°/s, and a deflection above 0.1°. The eye-tracking data consists of (*x, y*) gaze location entries for each individual time point (**Figures 3A, B**). Coordinates were given in pixels with respect to the monitor coordinates [the upper left corner of the screen was (0, 0) and down/right was positive]. We provide this raw data as well as various engineered eye-tracking features.

##### 2.6.1.2. Eye-tracking feature extraction

**Figure 2:**
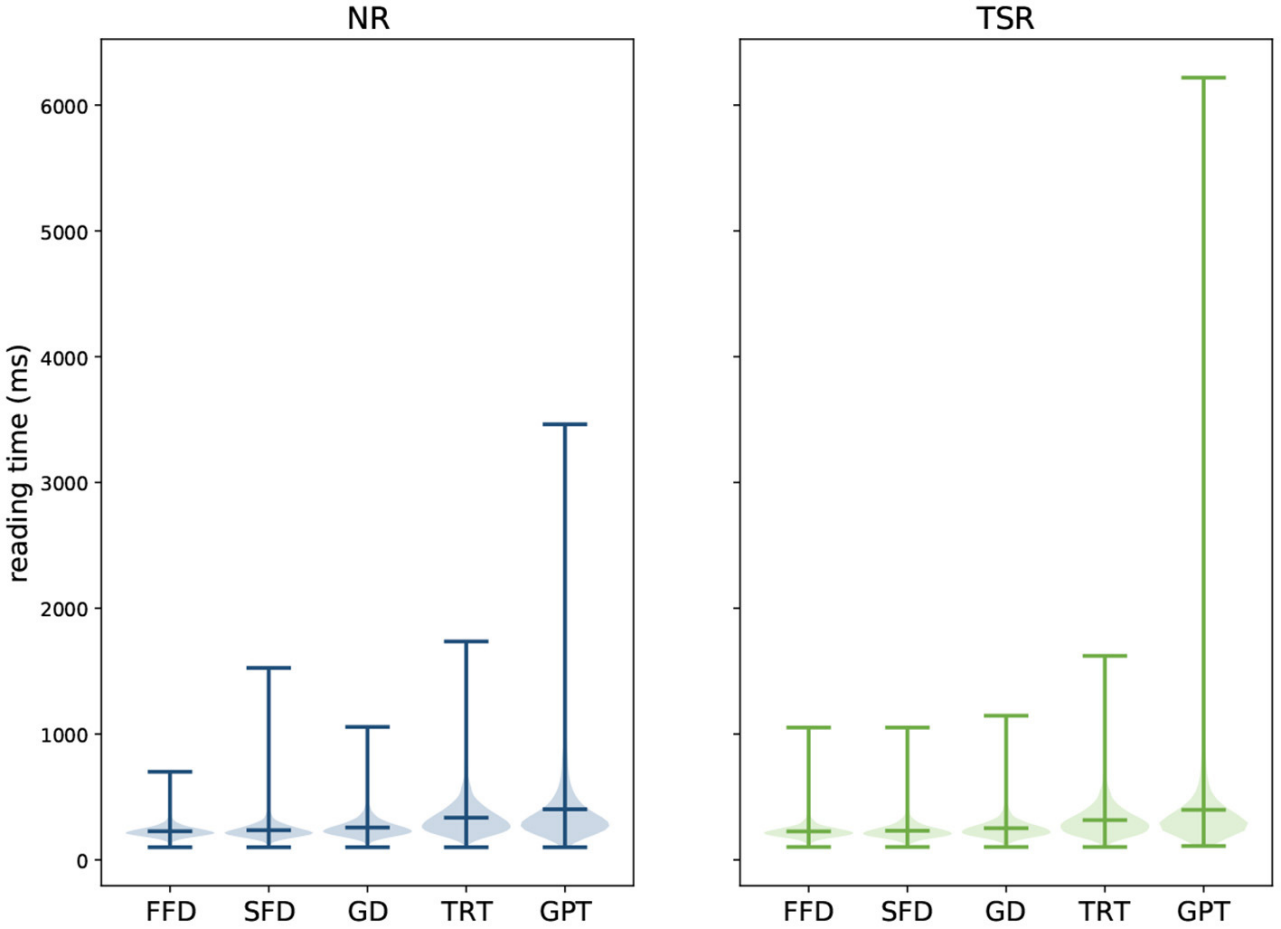
Violin plots showing means, distributions, and ranges of the reading time measures per word for each task and each eye-tracking feature (*x*-axis) in milliseconds.

For this feature extraction, only fixations within the boundaries of each displayed word were extracted. A Gaussian mixture model was trained on the (*y*-axis) gaze data for each sentence to improve the allocation of eye fixations to the corresponding text lines. The number of text lines determined the number of Gaussians to be fitted within the model. Subsequently, each gaze data point was clustered to the matching Gaussian and the data were realigned. As a result, each gaze data point is clearly assigned to a specific text line. Data points distinctly not associated with reading (minimum distance of 50 pixels to the text) were excluded. Additionally, fixations shorter than 100 ms were excluded from the analyses, because these are unlikely to reflect fixations relevant for reading (Sereno and Rayner, 2003). On the basis of previous eye-tracking corpora, namely the GECO corpus (Cop et al., 2017) and ZuCo 1.0 (Hollenstein et al., 2018), we extracted the following features: (i) *gaze duration* (GD), the sum of all fixations on the current word in the first-pass reading before the eye moves out of the word; (ii) *total reading time* (TRT), the sum of all fixation durations on the current word, including regressions; (iii) *first fixation duration* (FFD), the duration of the first fixation on the prevailing word; (iv) *single fixation duration* (SFD), the duration of the first and only fixation on the current word; and (v) *go-past time* (GPT), the sum of all fixations prior to progressing to the right of the current word, including regressions to previous words that originated from the current word. See Figure 2 for a visualization of the feature ranges of each reading task. For each of these eye-tracking features, we additionally computed the pupil size. Furthermore, we extracted the number of fixations and mean pupil size for each word and sentence. Additionally, on the sentence level, we extracted the mean and maximum saccade velocity, saccade amplitude and saccade duration. On the word level, saccade velocity, amplitude, and duration were extracted for in-going, outgoing, as well as saccades within a word. Finally, on the sentence level, omission rate is calculated, representing the proportion of words which were not fixated within each sentence.

#### 2.6.2. EEG preprocessing and feature extraction

##### 2.6.2.1. EEG preprocessing

Before the EEG preprocessing, data from all 14 blocks (seven NR and seven TSR) were first merged to avoid high predictive power based on the differences resulting from the preprocessing itself. To avoid loss of data by the subsequent automated preprocessing pipeline, the files of each recording blocked were screened to exclude highly artifactual data. Therefore, each block was temporarily filtered using a 2 Hz high-pass filter. Subsequently, outlying data points were removed if they exceeded a threshold of three standard deviations above or below the mean of the data. Only if the standard deviation of this temporarily pre-cleaned data was below a cut-off of 100 μV, the original corresponding block was used in the merging process. Applying this criterion, 4.02% of all blocks were excluded. The EEG preprocessing was conducted with the open-source MATLAB toolbox preprocessing pipeline Automagic (Pedroni et al., 2019), which combines state-of-the-art EEG preprocessing tools into a standardized and automated pipeline. The EEG preprocessing consisted of the following steps: First, bad channels were detected by the algorithms implemented in the EEGlab plugin clean_rawdata.^4^ A channel was defined as a bad electrode when recorded data from that electrode was correlated at <0.85 to an estimate based on other channels. Furthermore, a channel was defined as bad if it had more line noise relative to its signal than all other channels (four standard deviations). Finally, if a channel had a longer flat-line than 5 s, it was considered bad. These bad channels were automatically removed and later interpolated using a spherical spline interpolation (EEGLAB function eeg_interp.m). The interpolation was performed as a final step before the automatic quality assessment of the EEG files. Next, data were filtered using a 2 Hz high-pass filter and line noise artifacts were removed by applying Zapline (de Cheveigné, 2020), removing seven power line components. Subsequently, independent component analysis (ICA) was performed. Components reflecting artifactual activity were classified by the pre-trained classifier ICLabel (Pion-Tonachini et al., 2019). Components that were classified as any class of artifacts (line noise, channel noise, muscle activity, eye activity, and cardiac artifacts) with a probability higher than 0.8 were removed from the data. Subsequently, residual bad channels were excluded if their standard deviation exceeded a threshold of 25 μV. Very high transient artifacts (>100 μV) were excluded from calculating the standard deviation of each channel. However, if this resulted in a significant loss of channel data (>50%), the channel was removed from the data. After this, the pipeline automatically assessed the quality of the resulting EEG files based on four criteria: First, a data file was marked as bad-quality EEG and not included in the analysis if the proportion of high-amplitude data points in the signals (>30 μV) was larger than 0.20. Second, more than 20% of time points showed a variance larger than 15μ*V* across channels. Third, 30% of the channels showed high variance (>15 μV). Fourth, the ratio of bad channels was higher than 0.3. After Automagic preprocessing, 13 electrodes in the outermost circumference (chin and neck) were excluded from further processing as they capture little brain activity and mainly record muscular activity. The discarded electrode labels were E1, E8, E14, E17, E21, E25, E32, E48, E49, E56, E63, E68, E73, E81, E88, E94, E99, E107, E113, E119, E125, E126, E127, and E128. Additionally, 10 EOG electrodes were separated from the data and not used for further analysis, yielding a total number of 105 EEG electrodes. Subsequently, the data was converted to a common average reference.

##### 2.6.2.2. EEG and eye-tracking synchronization

**Figure 3:**
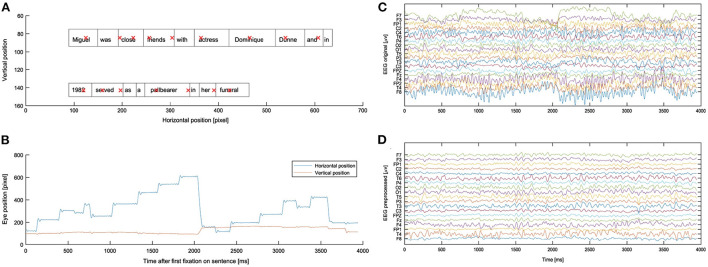
Visualization of eye-tracking and EEG data for a single sentence. **(A)** Prototypical sentence fixation data. Red crosses indicate fixations; boxes around the words indicate the wordbounds. **(B)** Fixation data plotted over time. **(C)** Raw EEG data during a single sentence. **(D)** Same data as in **(C)** after preprocessing.

In a next step, the EEG and eye-tracking data were synchronized using the “EYE-EEG” toolbox (Dimigen et al., 2011) to enable EEG analyses time-locked to the onsets of fixations and saccades, and subsequently segment the EEG data based on the eye-tracking measures. The synchronization algorithm first identified the “shared” events. Next, a linear function was fitted to the shared event latencies to refine the start- and end-event latency estimation in the eye tracker recording. Finally, the synchronization quality was ensured by comparing the trigger latencies recorded in the EEG and eye-tracker data. All synchronization errors did not exceed 2 ms (i.e., one data point). Remaining eye artifacts in data were removed with Unfold toolbox (Ehinger and Dimigen, 2019) according to a method described in Pfeiffer et al. (2020). The effect of this preprocessing can be seen from Figures 3C, D.

##### 2.6.2.3. EEG feature extraction

**Figure 4:**
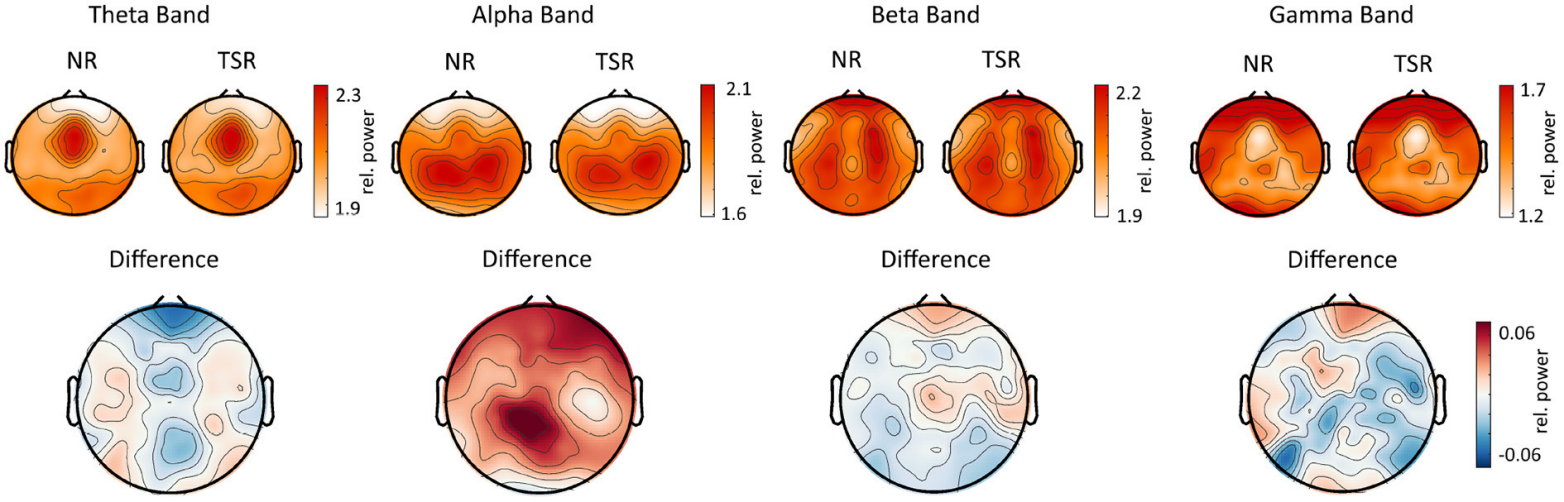
Topographical plots showing the mean EEG activity across all subjects from ZuCo 2.0. Averaged sentence level features are plotted in each reading condition as well as the difference between the tasks (NR minus TSR; scalp viewed from above, nose at the top). Only for the purpose of this visualization, relative power values are plotted (i.e., power in each frequency band divided by the average power between 1 and 50Hz), showing the expected typical power distribution across the scalp.

To compute oscillatory power measures, we band-pass filtered the continuous EEG signals across an entire reading task for four different frequency bands, resulting in a time-series for each frequency band. The distinct frequency bands were determined as follows: *theta_1* (4–6 Hz), *theta_2* (6.5–8 Hz), *alpha_1* (8.5–10 Hz), *alpha_2* (10.5–13 Hz), *beta_1* (13.5–18 Hz), *beta_2* (18.5–30 Hz), *gamma_1* (30.5–40 Hz), and *gamma_2* (40.5–49.5 Hz). Afterwards, we applied a Hilbert transformation to each of these time-series resulting in a complex time series. The Hilbert phase and amplitude estimation method yields results equivalent to sliding window Fourier transformation and wavelet approaches (Bruns, 2004). We chose specifically the Hilbert transformation to maintain temporal information for the amplitude of the frequency bands to enable the power computation of the different frequencies for time segments defined through fixations in the eye-tracking data. Finally, for each sentence as well as for each word within each sentence, and for each frequency band, the EEG features consist of a vector of 105 dimensions (one value for each EEG channel). On the level of individual words, these frequency band power features were calculated based on fixations of GD, TRT, FFD, SFD, and GPT (see above). For each EEG feature, all channels were subject to an artifact rejection criterion of 90 μV to exclude trials with transient noise. To descriptively compare the EEG activity and the extracted frequency band power between the NR and TSR sentences, the average of each condition as well as the differences (NR minus TSR) for the different sentence-level EEG features are plotted in Figure 4.

### 2.7. Data access

The raw and preprocessed EEG and eye-tracking data, as well as the features extracted from the preprocessed EEG and eye-tracking are provided for this benchmark. For the training data, the information about the task (normal reading or task-specific reading) is also available. Please note that for the held-out test dataset, we can only provide the preprocessed data and the extracted features. As the raw data were collected in different blocks of normal reading and task-specific reading, the participants could otherwise infer the outcome from the block separation. All the data can be accessed *via* OSF: https://osf.io/d7frw/.

## 3. Benchmark task

### 3.1. Task definition

We propose an ML benchmark for reading task identification. As described in Section 2, the ZuCo corpus provides data from two reading paradigms, normal reading (NR) and task-specific annotation reading (TSR). Consequently, we frame the problem as binary classification task with labels *Y* ∈ {NR, TSR}. The training data consists of sentences labeled depending on which reading task they belonged to during the experiment. Each sentence is represented by a feature set *X*. The input features should be eye-tracking or EEG features, or a combination thereof.

The goal of the benchmark task is to build a binary classifier *h* to predict the label *Y* for each sentence given only the features *X*:


(1) $$\begin{array}{l} h:X\to \{\text{NR},\text{TSR}\}. \end{array}$$

Due to the naturalistic experiment design and the co-registration of EEG and eye movement signals, feature extraction is possible on various levels. There are no restrictions to the type and dimension of the input features or the model.

### 3.2. Performance metrics

The classifier's performance is evaluated by the classification accuracy, defined as the number of correct predictions divided by the total number of predictions. Since previous results have shown high performance on models trained and tested within-subject but low performance on cross-subject models (Hollenstein et al., 2021c), this benchmark aims to address this gap by focusing on the latter to improve the inter-subject generalization capabilities of the models. We propose a cross-subject evaluation, where each subject in the held-out testset is evaluated by a model trained on all subjects in the training split (i.e., the original ZuCo 2.0 dataset). Therefore, the main benchmark metric is defined as the mean classification accuracy across all subjects in the testset. As a second metric, we choose the F1-measure. In our classification setup, we do not distinguish between a positive and a negative class, i.e., there is no clear majority or minority class. For that reason, we choose to evaluate our classifier using the macro-averaged F1-scores. The benchmark task is evaluated on models from the following three categories: models trained on EEG features, models trained on eye-tracking features, and models trained on a combination of EEG and eye-tracking features.

### 3.3. Benchmark setup

We host the ZuCo benchmark on Eval-AI (Yadav et al., 2019) – an open source AI challenge platform for evaluating and comparing machine learning and artificial intelligence algorithms. The link to the reading task classification challenge and more information on how to participate is available here: https://github.com/norahollenstein/zuco-benchmark. This solution will help other researchers to participate in our machine learning challenge and enable us to automate the evaluation of the future submissions.

#### 3.3.1. Evaluation strategy

Researchers that want to participant in the benchmark task can submit predictions from their models for the hidden testset. We specified the challenge configuration, evaluation code, and information about the data splits. Predictions for the testset labels can be submitted in the JSON file.

#### 3.3.2. Leaderboard

The public leaderboard will include the scores on the chosen evaluation metrics as well as references to upcoming publications. Upon submission, the predictions will be handed over to challenge-specific workers that compare the predictions against corresponding ground-truth labels using the custom evaluation script provided by our team.

## 4. Baseline methods

### 4.1. Textual baselines

We set three minimal baselines for this benchmark task: (i) a random baseline, (ii) a word embedding baseline, and (iii) a text difficulty baseline. We will use the first one as the basis for model comparison, while the latter two serve merely as control conditions to validate the dataset and exclude linguistic properties as a possible confound in the reading task classification benchmark.

#### 4.1.1. Random baseline

We compute a random baseline to assess the chance level of predicting the correct class. We randomly sample the labels according to the distribution of the training data. That means the label NR is chosen with a probability of $p_{NR}=\frac{390}{739}\approx 0.53$ and TSR is chosen with *p*_*TSR*_ = 1−*p*_*NR*_ ≈ 0.47.

#### 4.1.2. Word embedding baseline

Even though the experimental design of ZuCo ensured the similarilty of the sentences in terms of sentence lengths and text complexity, we aim to ensure the sentences in the data are not easily separable merely by their linguistic characteristics. Therefore, we compare our models to a textual baseline as a sanity check. For this purpose, we use pre-trained textual representations, namely, the state-of-the-art contextualized BERT word embeddings (Devlin et al., 2019). We concatenate the embeddings of all words in a sentence and feed them into the LSTM model.

#### 4.1.3. Text difficulty baseline

**Figure 5:**
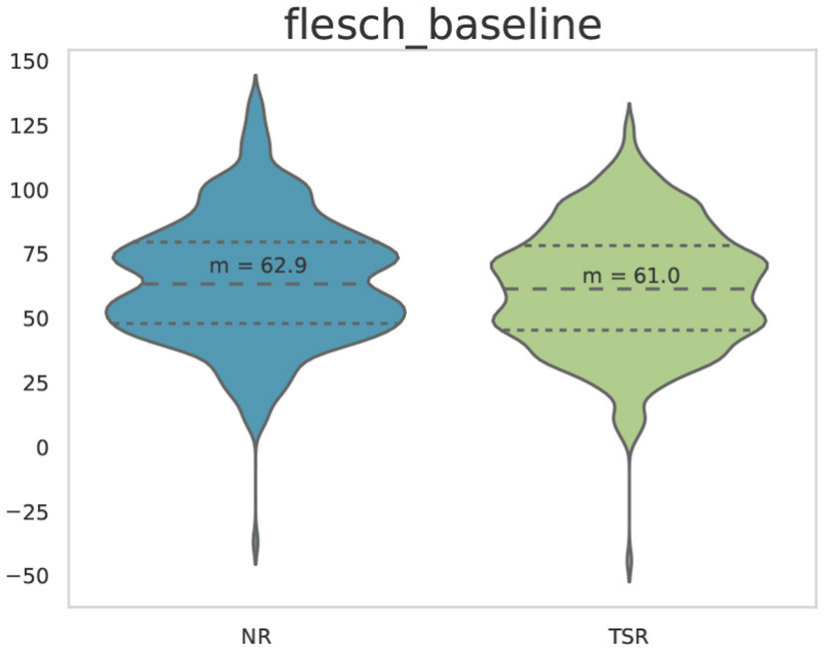
Flesch reading ease (FRE) scores for the NR and TSR sentences used in the ZuCo 2.0. dataset.

We also provide a baseline based on text readability. Although the sentences for both reading tasks were chosen to be of similar length and from the same text genre, we want to ensure that both tasks are not separable merely by the difficulty of the sentences. Therefore, we implement a text difficulty baseline, which classifies the sentences into NR and TSR based on their Flesch reading ease score (FRE; Flesch, 1948). This score indicates how difficult an English text passage is to understand based on the average number of words in a sentence and the average number of syllables in a word:


(2) $$\begin{array}{l} FRE=x-y\left(\frac{words}{sentences}\right)-z\left(\frac{syllables}{words}\right) \end{array}$$

where *x*, *y* and *z* are language-specific weighting factors (for English *x* = 206.835, *y* = 1.015, *z* = 84.6). We compute FRE scores for each of the English sentences in the ZuCo data. Figure 5 shows the distribution of the FRE across the sentences of ZuCo 2.0.

### 4.2. EEG and eye-tracking models

We also present a set of initial models using EEG and eye-tracking features as a starting point for future models.^5^ For each sentence in the dataset, the model input is composed of a vector of eye-tracking and/or EEG features corresponding to a single sentence in the dataset. Each sample in the training set is labeled with the reading task it was recorded in, normal reading (NR) or task-specific reading (TSR). We investigate the potential of using sentence-level eye-tracking and EEG features for the reading task classification. Hollenstein et al. (2021c) compared sentence-level and word-level features for this task previously and showed that sentence-level features perform better. However, challenge participants are also invited to use word-level and other features (see discussion in Section 6 for suggestions). The advantages of sentence-level features consist of the possibility of using simpler machine learning models and reduced training times (Hollenstein et al., 2021c). Sentence-level features are defined as metrics aggregated over all words in a given sentence.

#### 4.2.1. Eye-tracking features

We include two types of sentence-level eye-tracking features. The features are summarized in Table 5. First, the fixation-based features - omission rate, number of fixations and reading speed - are aggregated metrics normalized by sentence length, i.e., the number of words in a sentence. Analogous to the word-level models, we also include saccade-based features. These include the mean and maximum duration, velocity and amplitude across all saccades that occurred within the reading time of a give sentence. We test these features individually and combined to investigate the performance increase achieved by adding more features.

**Table 5:** Sentence-level eye-tracking features.

**Name**	**Definition**	**Values**
**Fixation features**		
omission_rate	Percentage of words in a sentence that is *not* fixated	1
fixation_number	Number of fixations in the sentence divided by the number of words	1
reading_speed	Sum of the duration of all fixations in the sentence divided by the number of words	1
**Saccade features**		
mean_sacc_dur	Sum of the duration of all saccades in the sentence divided by the number of words	1
max_sacc_dur	Maximum saccade duration per sentence	1
mean_sacc_velocity	Sum of the velocity of all saccades in the sentence divided by the number of saccades	1
max_sacc_velocity	Maximum saccade velocity per sentence	1
mean_sacc_amplitude	Sum of the amplitude of all saccades in
the sentence divided by the number of saccades		1
max_sacc_amplitude	Maximum saccade amplitude per sentence	1
**Combined features**		
Combined ET features	Concatenation of all eye-tracking features	9

We use the combination of all features for our models.

#### 4.2.2. EEG features

The sentence-level EEG features take into account the EEG activity over the whole sentence duration (even when no words were fixated). We aggregate over the preprocessed EEG signals of the full reading duration of a sentence. Each subfrequency band (e.g., *alpha_1* and *alpha_2*) were averaged to get one power measure for each frequency band, i.e., *theta* (4–8 Hz), *alpha* (8.5–13 Hz), *beta* (13.5–30 Hz), and *gamma* (30.5–49.5 Hz). The sentence-level EEG features are described in Table 6. We experiment with both aggregate metrics, i.e., the mean across all electrodes, and individual electrode features.

**Table 6:** Sentence-level EEG features.

**Name**	**Definition**	**Values**
**Mean features**		
theta_mean	Mean theta band features averaged over all electrodes	1
alpha_mean	Mean alpha band features averaged over all electrodes	1
beta_mean	Mean beta band features averaged over all electrodes	1
gamma_mean	Mean gamma band features averaged over all electrodes	1
eeg_means	Mean frequency band features averaged over all electrodes, resulting in 1 feature value for each of the 8 frequency bands	8
**Electrode features**		
electrode_features_theta	Mean theta1 and theta2 values of all 105 electrodes	105
electrode_features_alpha	Mean alpha_1 and alpha_1 values of all 105 electrodes	105
electrode_features_beta	Mean beta_1 and beta_1 values of all 105 electrodes	105
electrode_features_gamma	Mean gamma_1 and gamma_1 values of all 105 electrodes	105
electrode_features_all	Concatenation of the four features above	420
**Combined features**		
ET & EEG mean features	Concatenation of sent_gaze_sacc and eeg_means	17

**Figure 6:**
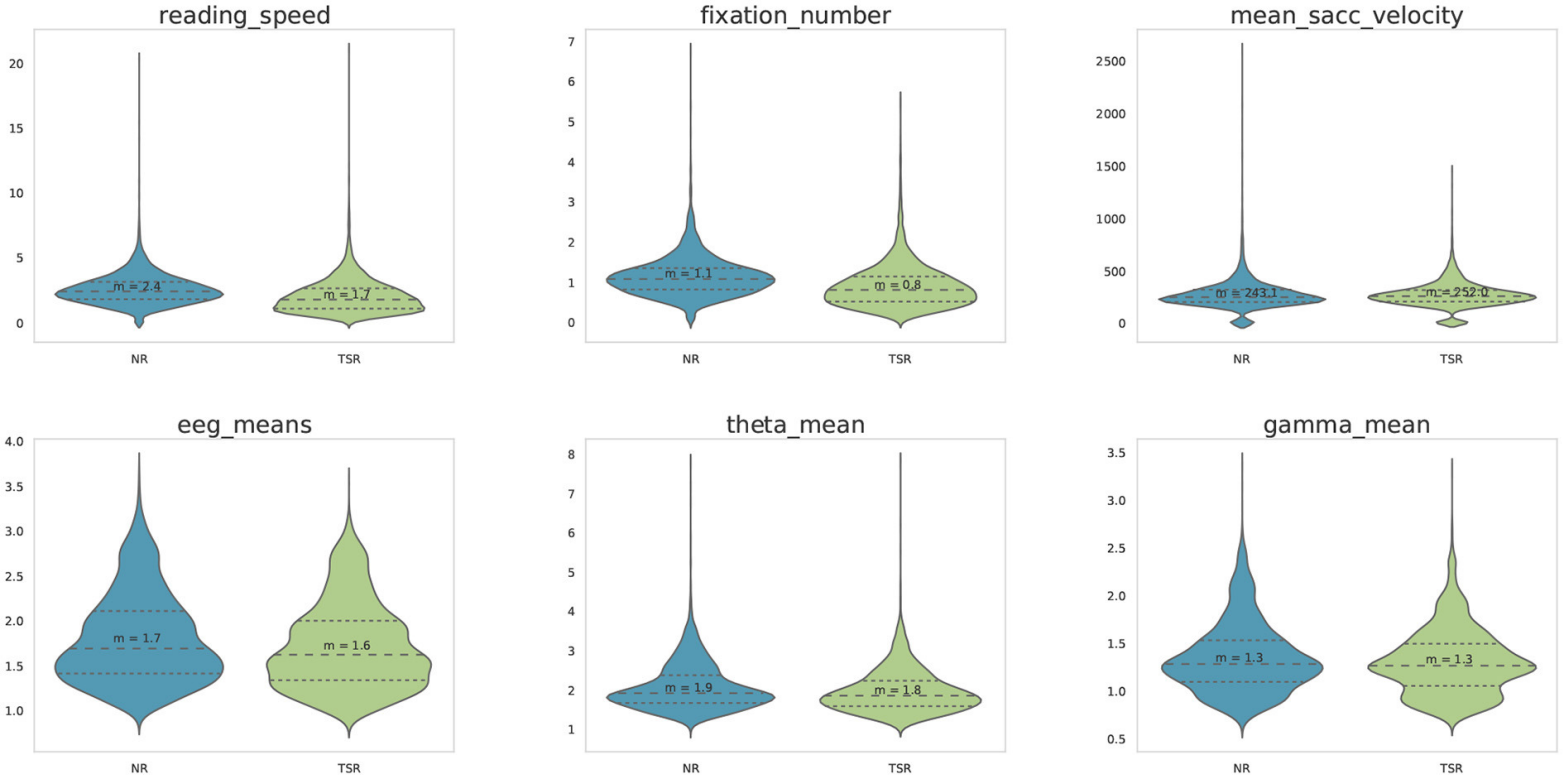
Examples of feature distributions across all subjects for the NR and TSR sentences included in the ZuCo 2.0. dataset.

Examples of these features across all subjects, split by class (normal reading vs. task-specific reading) are shown in Figure 6 for ZuCo 2.0.

#### 4.2.3. Principal component analysis

**Figure 7:**
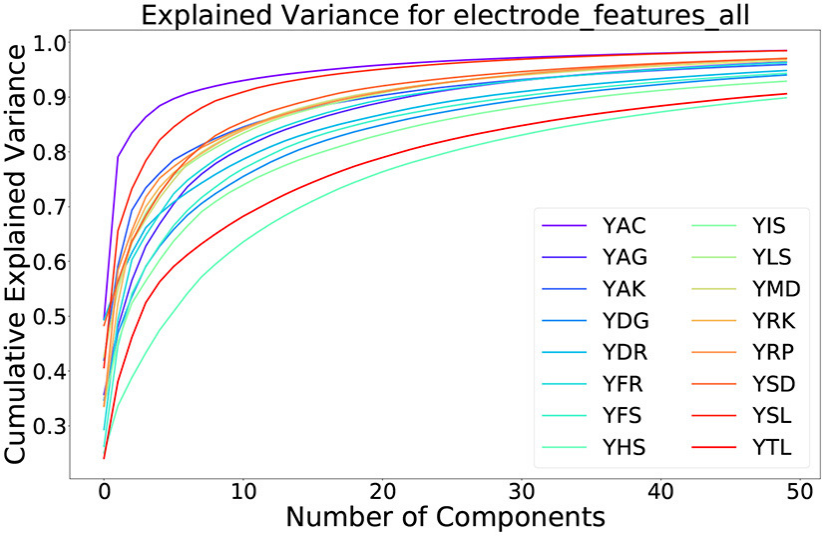
Variance explained with increasing number of PCA components for the training subjects in ZuCo 2.0.

We use principal component analysis (PCA) to reduce the dimensionality of the EEG features. In an initial attempt, we fitted PCA on all training subjects and applied it to both the training and test split. This, however, led to no significant improvements in classification accuracy. Thus, we fit PCA to each subject individually. To prevent overfitting to the test subjects, we only consider subjects in the training data to determine the number of components. We fit PCA for each subject separately and calculate the number of components that explain 95% of the variance. We then choose the number of components of PCA as the median over all subjects in the training data, which makes it robust against outlier subjects. The result is a reduced dimensionality from 105 to 41 of both training and test data. Figure 7 shows that the amount of variance explained by the first components varies significantly between subjects. The first component, for instance, accounts for ~24% of the variance for subject YTL, whereas it accounts for 49% of the variance for subject YAC.

To analyze how much the individual electrodes influence the principal components, we again fit PCA for each subject of the training data, such that the resulting components explain 95% of the variance. Assuming we have *n* original features and *m* principal components *c*, where each component is a linear combination of the original features, i.e., $c^{j}=\overset{n}{\underset{i}{\sum }}\beta_{i}^{j}x_{i},j\in 1\ldots m$. We then extract the amount of variance explained (*v*^*j*^) by each component *c*^*j*^ and its weights $\beta_{i}^{j}$. We sum up all $\beta_{i}^{j}$ weighted by *v*^*j*^, such that the resulting $\beta_{i}=\overset{m}{\underset{j}{\sum }}v^{j}\beta_{i}^{j}$ represents the relevance of feature *x*_*i*_.

**Figure 8:**
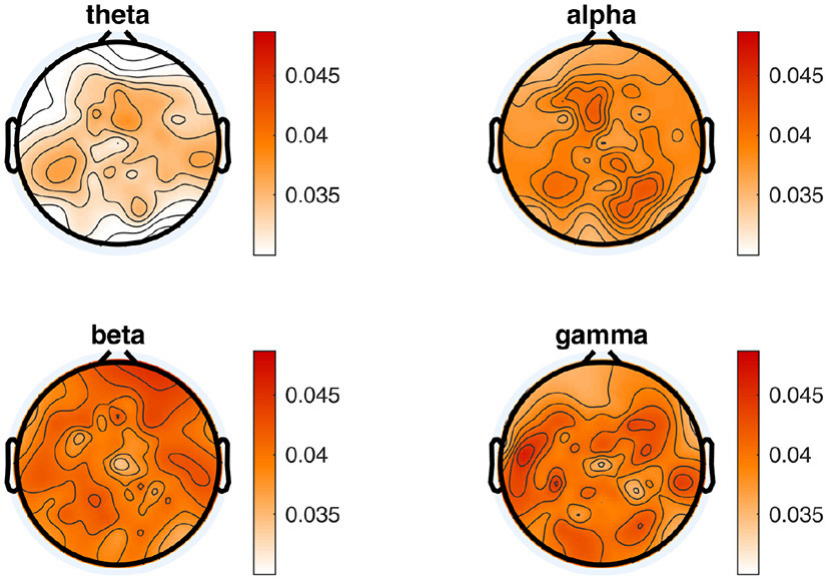
Topographical distribution of electrode importance for the principle components, divided into the 4 different frequency bands. Electrode importance is calculated by determining the influence of each electrode on the principle components and weighting them by amount of explained variance.

Following this procedure, we split the results into frequency bands and present the corresponding topography plots averaged over all training subjects in ZuCo 2.0 in Figure 8.

#### 4.2.4. Model

The input to the sentence-level model is a single vector representing each sentence. We scale the feature values to a range between {0, 1}. We train a support vector machine for classification with a linear kernel. We use the scikit-learn SVC implementation.^6^ For the cross-subject evaluation, the models are trained on all samples from all subjects in ZuCo 2.0 and tested on the samples from new subjects in the held-out testset.

## 5. Results

### 5.1. Results of textual baselines

As described in the previous section, we set three minimal baselines for this benchmark task: (i) a random baseline, i.e., chance level for binary classification, (ii) a word embedding baseline, namely BERT word embeddings, and (iii) a text difficulty baseline, based on the Flesch reading ease score (FRE). The random baseline for binary classification is at 0.50 accuracy. The word embedding baseline yield a classification accuracy of 0.65 for ZuCo 2.0. The text difficulty baseline is also above random performance with a classification accuracy of 0.53 for ZuCo 2.0. Table 7 shows the accuracy and F1-score for all baselines.

**Table 7:** The mean accuracy and F1-score over all subjects for each feature-set in the benchmark task.

**Feature set**	**Accuracy**	**F1**
Random	0.50	0.50
FRE baseline	0.53	0.35
BERT baseline	0.65	0.64
Eye-tracking features	0.69	0.67
Eye-tracking and EEG mean features	0.68	0.66
Concatenated EEG electrode features	0.55	0.46
Concatenated EEG electrode features (with PCA)	0.58	0.56

### 5.2. Results of EEG and eye-tracking models

As described in Section 3, we consider three different feature sets, EEG, eye-tracking, and the combination of all features. For each feature set and each subject, we report the accuracy and the F1-score. For each subject in the hidden testset, we compute the results *via* bootstrapping, sampling 500 times with replacement, and using a sample size equal to the original data. For all results, we report the comparison to the random and textual baselines as well as the 95% confidence intervals for each subject. Table 7 shows a summary of the results. The corresponding tables with the detailed numbers for all subjects and feature sets are shown in Appendix 1.

**Figure 9:**
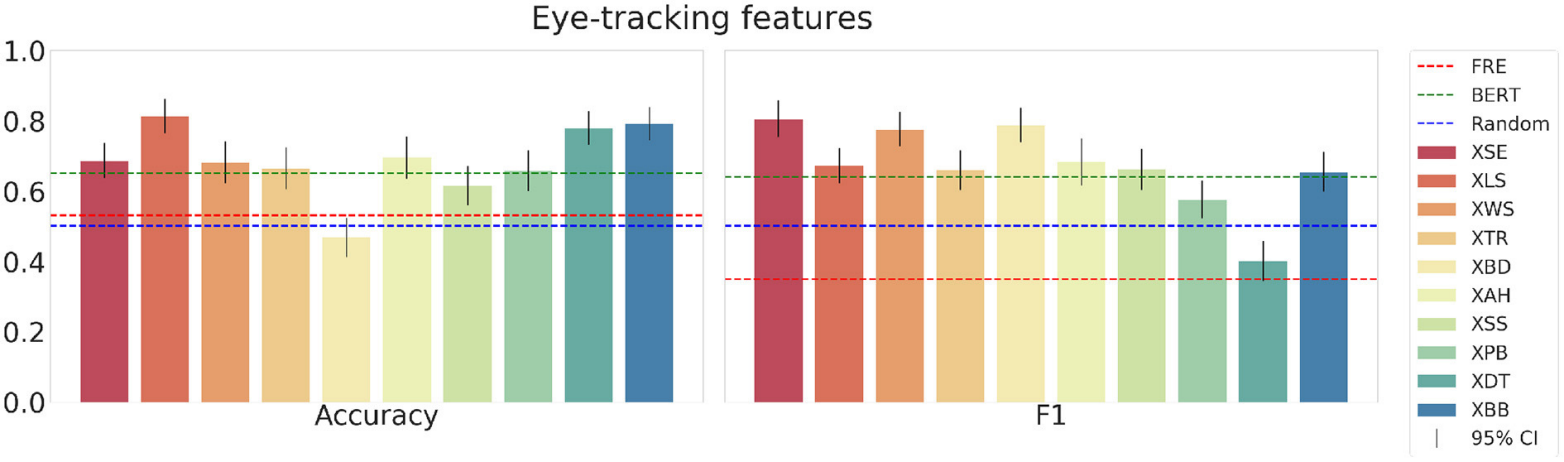
The mean accuracy **(left)**, mean F1-score **(right)** with corresponding 95% confidence intervals and textual baselines are plotted for each subject in the held-out test dataset using the concatenated eye-tracking features.

**Figure 10:**
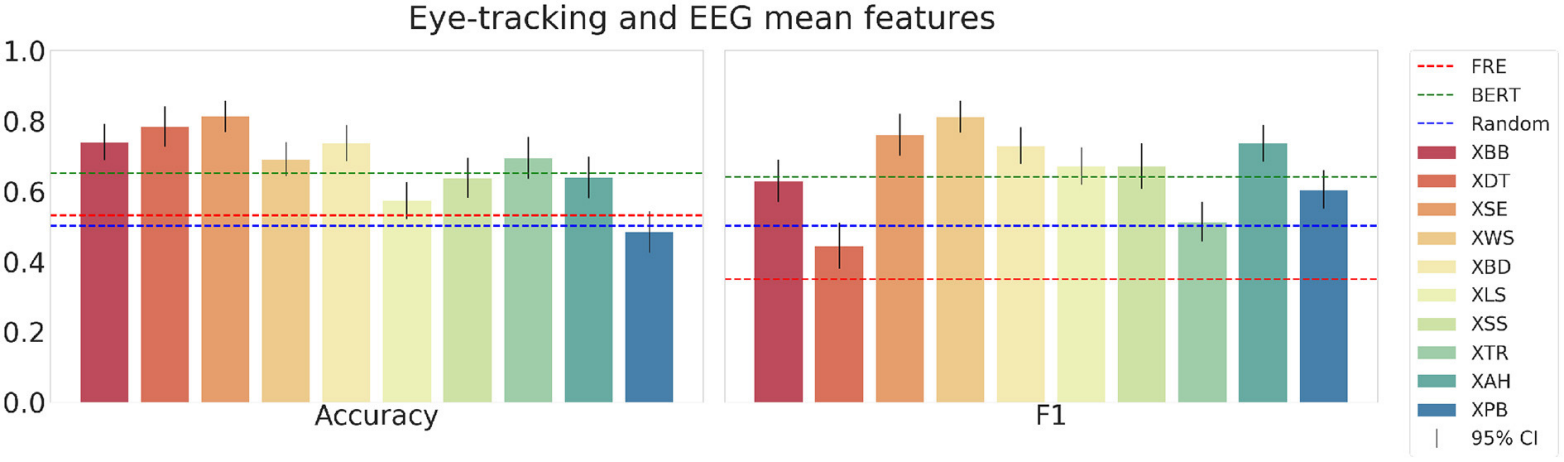
The mean accuracy **(left)**, mean F1-score **(right)** with corresponding 95% confidence intervals and textual baselines are plotted for each subject in the held-out test dataset using the eye-tracking and EEG mean features.

First, the results for the eye-tracking features are shown in Figure 9. These results clearly show all subjects outperforming the random baseline and FRE control model except for one subject each for accuracy and F1-score. All subjects except one perform better than the random baseline, and three subjects perform significantly better than the BERT word embedding control model. The mean accuracy across all subjects in the testset is 0.69, and the mean F1-score is 0.67. Furthermore, the results for the combined eye-tracking and EEG mean feature set in Figure 10 do not yield an increase in performance compared to using only the eye-tracking features (mean accuracy: 0.68; F1-score: 0.66). Interestingly, the best and worst performing subjects vary between different feature combinations, and between accuracy and F1-score.

**Figure 11:**
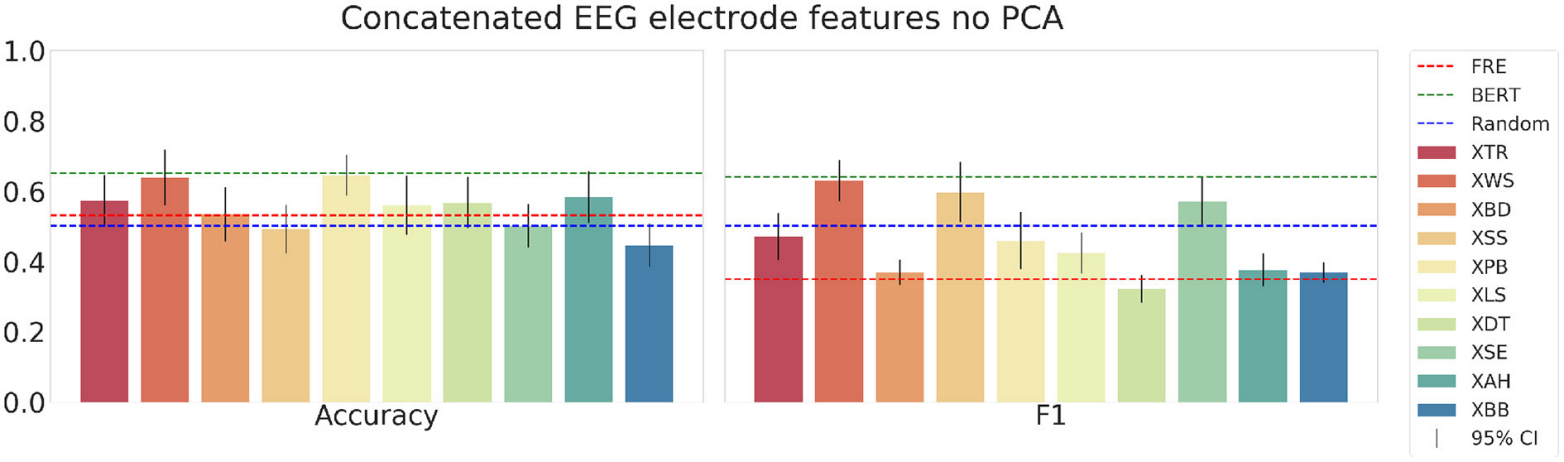
The mean accuracy **(left)**, mean F1-score **(right)** with corresponding 95% confidence intervals and textual baselines are plotted for each subject in the held-out test dataset using the concatenated EEG electrode features without PCA pre-processing.

**Figure 12:**
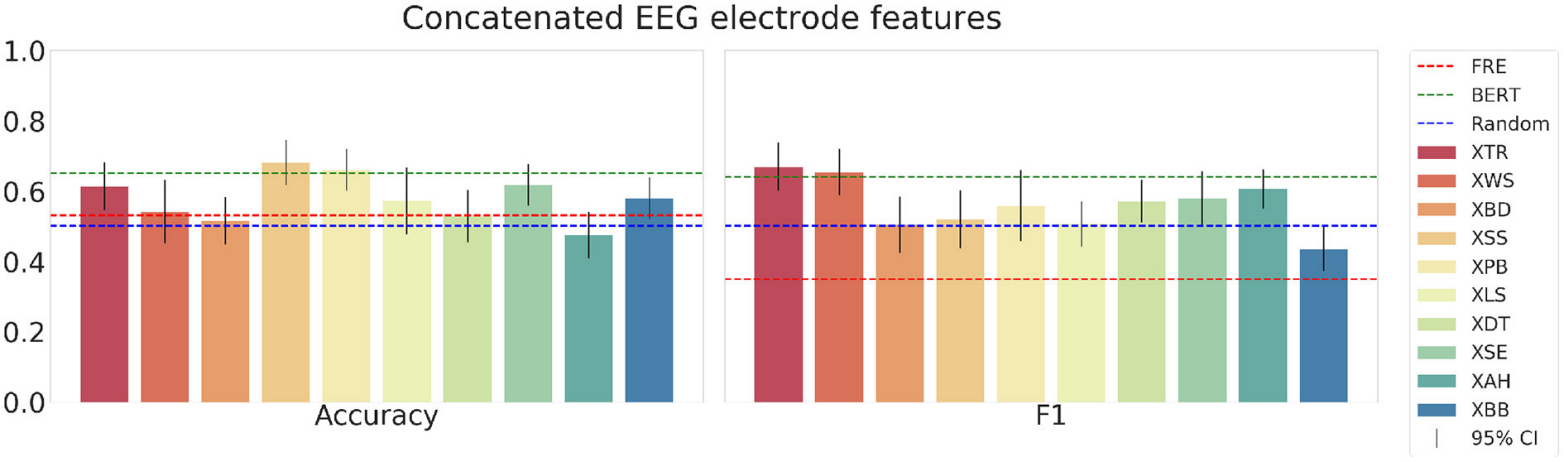
The mean accuracy **(left)** mean, F1-score **(right)** with corresponding 95% confidence intervals and textual baselines are plotted for each subject in the held-out test dataset using the concatenated EEG electrode features after pre-processing with PCA.

Next, we show the results using the concatenated EEG electrode features^7^ in Figure 11. With this feature set, the mean accuracy across all subjects in the testset is 0.55, and the mean F1-score is 0.46. The accuracy scores are notably higher than for the F1-score. Finally, when using the same features but applying the PCA preprocessing, the models yield the results presented in Figure 12. The scores for the accuracy are similar but have a slightly higher mean of 0.58 (compared to 0.55 without PCA). However, the F1-scores with PCA are significantly higher with a mean of 0.56 (compared to 0.46 without PCA). While with these EEG electrode features the models outperform the random and text difficulty baseline for some test subjects, they do not achieve to outperform the strong embedding baseline. Additionally, we experimented with combining the BERT embeddings with the EEG and eye-tracking feature sets in the SVM models. However, the combination of linguistic and physiological features did not yield any improvements.

## 6. Discussion

The present benchmark challenge has the main goal of advancing reading task classification through eye-tracking and EEG data. The challenge participants are invited to develop ML models to identify whether subjects are reading a sentence with the goal of reading comprehension (i.e., normal reading) or whether the subjects are reading a sentence to search for a specific semantic relation in the sentence (i.e., task-specific reading). The objective is to investigate which eye movement and brain activity features are most suited to solve this problem. Understanding the physiological aspects of the reading process (i.e., the cognitive load and reading intent) can advance our understanding of human language processing and general attentional processes. On the other hand, natural language processing and machine learning would benefit, as classifiers that outperform current textual baselines could improve the quality and process of collecting annotated data (e.g., through gaze-aided unsupervised labeling).

Several previous studies have used ML models to accurately perform a reading task classification. Cole et al. (2011) used eye-tracking data to discriminate between a scanning task and a reading comprehension task. Furthermore, Biedert et al. (2012) developed a real-time classifier able to distinguish reading from skimming patterns. In a related study, Kelton et al. (2019) investigated the influence of different content and tasks on the performance to determine whether subjects are reading or skimming a news article. Other neuroimaging methods such as fMRI have been combined with eye-tracking to examine the neural basis of sentence comprehension (e.g., Bonhage et al., 2015) or the discrimination between normal and non-word text (Choi et al., 2014). In another fMRI study, which simultaneously recorded eye-tracking data, Ceh et al. (2021) observed that internally and externally directed cognition are characterized by distinct brain activity. In addition, several research groups provide publicly available fMRI data to study naturalistic reading comprehension (Dehghani et al., 2017; Lopopolo et al., 2018; Pereira et al., 2018; Shain et al., 2020; Nastase et al., 2021). While functional MRI has a better spatial resolution compared to EEG, is a very costly method with restricted real-life usability. Whereas eye-tracking and EEG systems are of lower cost and can be used in more naturalistic situations. Several other publicly datasets recorded eye-tracking (e.g., Cop et al., 2017; Luke and Christianson, 2018; Jäger et al., 2021) or EEG from continuous speech stimuli (e.g., Broderick et al., 2018; Brennan and Hale, 2019). These datasets provide the possibility to improve and evaluate machine learning systems for NLP. However, to the best of our knowledge, the ZuCo dataset is the largest publicly available dataset that features simultaneous eye movement and EEG data recorded in a naturalistic reading setup. One recent addition is the CoCoNUt dataset by Frank and Aumeistere (2022), which contains 200 Dutch sentences with combined EEG and eye-tracking recordings. However, the selection of sentences is not completely natural, as it is guided by sentence length and word frequency. Thus, ZuCo is specifically tailored to leverage EEG and eye-tracking data to improve natural language processing tasks in a naturalistic setting. The field of machine learning contains a range of tasks on different modalities such as language (text), computer vision (video, images), and speech recognition (audio). Recently, Akbari et al. (2021) have shown superior performance of ML models with multimodal representations on downstream tasks such as image classification. Therefore, from an NLP perspective, another extension to this benchmark could be to investigate whether leveraging multimodal embeddings is beneficial for reading task classification.

In a recent study, the ZuCo data has been used already for reading task identification (Mathur et al., 2021) using a complex convolutional network, which is evaluated on a fixed cross-subject scenario on the sentences from ZuCo 2.0. However, the relatively poor performance of their model evaluated in a fixed cross-subject scenario, still leaves room for improvement and opens research questions regarding the selection of features. Hollenstein et al. (2021c) have recently presented extensive work on reading task classification, corroborating the advantages of the ZuCo dataset for this ML task. The authors found that, while high accuracy can be achieved on within-subject models, the performance drops for cross-subject evaluations. There is clearly room for improvement in the performance of the results presented in this work. However, these are still very promising results considering the complex nature of human physiological data.

A current bottleneck in machine learning is the lack of generalization capabilities of these models, meaning that the models perform poorly on data from other domains that are not included in their training data. For instance, ML models perform less accurately across languages, across image or text domains, or across subjects. The latter is of great importance in neuroscientific research which aims at a principled understanding of human brain activity as a response to complex stimuli (Nastase et al., 2019), as well as for practical applications such as brain-computer interfaces (Chiang et al., 2019). Specifically, when trained on physiological data, the rules identified by ML models for a given task ideally hold for the entire population. Considering the ever-increasing complexity of ML models due to their large number of parameters, they are prone to overfit to their training set (which does not characterize the entire population), leading to spurious correlations. Therefore, to validate the gained insights on the physiological data, ML models need to be evaluated on held-out subjects as a proxy to the model's generalization capability. These results inspired the proposed benchmark based on the ZuCo dataset. The benchmark task and baseline models follow the rules suggested by Scheinost et al. (2019) to take into account subject-specific differences in predictive modeling.

In the current paper, we provide evidence that both eye-tracking and brain activity data can improve reading task classification compared to purely text-based baselines. The best-performing model is based on sentence-level eye-tracking features. Combining eye-tracking and EEG mean features yields promising results, but not better than only eye-tracking features. One explanation for this is that the combination of eye-tracking and EEG features decreases the signal-to-noise ratio even more than for only one type of cognitive processing signal. Another explanation is that the eye-tracking and EEG signals contain redundant information. This is always a risk when using co-registered data of EEG and eye-tracking signals within the same task. Specifically, eye movement artifacts could be contained in the EEG data. However, in this work, we use state-of-the-art methods to remove eye movement artifacts in the EEG data (through ICA and Unfold). In short, there are possible gains in performance to be achieved by more sophisticated combinations of eye movement and brain activity features.

There are various ways to leverage eye-tracking and EEG data. Currently, we extracted high-level eye-tracking features based on fixations (e.g, number of fixations and omission rate) and on saccades (e.g., mean velocity and maximum amplitude). The ZuCo dataset provides additional reading-related features such as mean fixation duration, total reading time or go-past time, but also pupil size information or even the raw data could be used in future approaches. Using raw data has shown great promise to model eye-tracking data (e.g., Jäger et al., 2020), and one of the main advantages of the ZuCo dataset is that it allows feature extraction on different levels. Moreover, our EEG features include mean features aggregated over all electrodes as well as electrode-based frequency measures, which have been shown to improve NLP tasks in the past (Hollenstein et al., 2019a, 2021b; Sun et al., 2020; Wang and Ji, 2021). Nonetheless, we want to highlight that preprocessed EEG data permits the examination of additional measures, such as source-level based features (e.g., source-level power estimates) and functional connectivity measures at the level of the underlying neuronal generators. Other EEG analysis methods allow the extract measures of spatio-temporal dynamics of brain activity (e.g., microstates) (Michel and Koenig, 2018) and event-related potentials such as N400 components (Frank et al., 2013; Brouwer et al., 2017). Interestingly, Hollenstein et al. (2021c) found that gamma band features worked best in a within-subject setting. However, we found that concatenating all EEG electrode features is more beneficial in a cross-subject setting. Finally, the cross-subject performance can be further increased by using a dimensionality reduction (PCA) on the concatenated EEG features. Future methods could focus on new approaches for EEG feature selection and aggregation.

The simultaneous recording of EEG and eye-tracking allows us to investigate specific feature sets on different levels of analysis, e.g., sentence level, word level, fixation level. Nevertheless, one should note that the ZuCo dataset includes reading individual sentences rather than full document, which influences the reading behavior. Reading studies with longer text spans should be considered in future work. Additionally, the naturalistic setup of the experiments used in this work are crucial for this benchmark task and for neuroscience in general (Nastase et al., 2020). Not only does it increase the ecological validity of the recordings by allowing natural reading without controlling the individual reading speed, but it also supports the extraction of signals on various linguistic levels (Hasson and Egidi, 2015; Brennan, 2016; Alday, 2019; Kandylaki and Bornkessel-Schlesewsky, 2019; Hamilton and Huth, 2020). Frey et al. (2018) investigated how two different reading tasks modulate both eye movements and brain activity. In line with our findings, their results show that eye movement patterns were top-down modulated by different task demands. Moreover, their brain activity analysis suggests that the decision-making process during task-specific reading elicits a greater load in working memory than the one generated in a normal reading task. In summary, eye-tracking and EEG data offer an immensely diverse amount of potential measures, which might contain unique valuable information. Thus, we aim to inspire benchmark challenge participants to explore and extract alternative features from the available preprocessed data.

## 7. Conclusion

We presented a new ML benchmark using eye-tracking and EEG data to classify reading tasks. The goal of the benchmark challenge is to distinguish between normal reading and task-specific reading in a cross-subject evaluation scenario. We provide multiple initial models for this task and show that ML models trained on eye-tracking and EEG features can outperform strong textual baselines.

The standardized Zurich Cognitive Language Processing Corpus (ZuCo) dataset facilitates the creation of such a machine learning benchmark. We use the ZuCo 2.0 dataset as training data. To make our benchmark task more robust, we have additionally recorded further eye-tracking and EEG data from natural reading from additional subjects in a hidden testset. ZuCo's rich structure and high-density coverage of simultaneous EEG and eye-tracking signals can also help to advance other areas that study the combination of gaze position and brain activity to identify variations in attention, reading patterns and reading intents, as well as participants' compliance with the task demands and cross-subject variability.

Our dataset and benchmark setup allows us to easily add additional machine learning tasks to the leaderboard in the future. For instance, we can add additional NLP tasks since the ZuCo datasets provide ground truth labels for sentiment analysis or relation detection from text. Additionally, adding tasks such as eye movement and ERP prediction would be beneficial for various research communities. For example, the prediction of eye movement patterns has gained interest also in the NLP community (Hollenstein et al., 2021a). The main goal of this work is to create a platform for discussion and future research on a common benchmark task for reading task classification based on eye movement and brain activity data. We hope that this benchmark allows other researchers to make progress in this interdisciplinary research field.

## Data availability statement

The datasets presented in this study can be found in online repositories. The names of the repository/repositories and accession number(s) can be found in the article/ Supplementary material.

## Ethics statement

The studies involving human participants were reviewed and approved by Ethics commission of the University of Zurich. The patients/participants provided their written informed consent to participate in this study.

## Author contributions

NH: lead author, data collection, writing and editing, and machine learning. MT and MP: neuroscience experts, writing and editing, preprocessing and data analysis, and data collection. SK and YÖ: machine learning experts, writing and editing, and preprocessing and data analysis. LJ and NL: PIs and writing and editing. All authors contributed to the article and approved the submitted version.

## Footnotes

^1^Benchmark data available here: https://osf.io/d7frw/.

^2^Code for baseline methods available here: https://github.com/norahollenstein/zuco-benchmark.

^3^Data available here: https://osf.io/q3zws/.

^4^ http://sccn.ucsd.edu/wiki/Plugin_list_process

^5^The code is available here: https://github.com/norahollenstein/zuco-benchmark.

^6^ https://scikit-learn.org/stable/modules/generated/sklearn.svm.SVC.html

^7^These figures show the results for absolute EEG power. The results for relative EEG power are depicted in the Appendix 1 in Figure 13.

## References

[B1] Abdelrahman Y. Khan A. A. Newn J. Velloso E. Safwat S. A. Bailey J.. (2019). “Classifying attention types with thermal imaging and eye tracking,” in Proceedings of the ACM on Interactive, Mobile, Wearable and Ubiquitous Technologies, Vol. 3 (New York, NY: Association for Computing Machinery), 1–27. 10.1145/335122734164595

[B2] Akbari H. Yuan L. Qian R. Chuang W.-H. Chang S.-F. Cui Y.. (2021). VATT: transformers for multimodal self-supervised learning from raw video, audio and text. arXiv. [preprint]. 10.48550/arXiv.2104.11178

[B3] Alday P. M. (2019). M/EEG analysis of naturalistic stories: a review from speech to language processing. Lang. Cogn. Neurosci. 34, 457–473. 10.1080/23273798.2018.1546882

[B4] Barrett M. Bingel J. Hollenstein N. Rei M. Søgaard A. (2018). “Sequence classification with human attention,” in Proceedings of the 22nd Conference on Computational Natural Language Learning (Brussels, Belgium: Association for Computational Linguistics), 302–312. 10.18653/v1/K18-1030

[B5] Bautista L. G. Naval P. (2020). “Towards learning to read like humans,” in International Conference on Computational Collective Intelligence (New York, NY: Springer), 779–791. 10.1007/978-3-030-63007-2_61

[B6] Bestgen Y. (2021). “LAST at CMCL 2021 shared task: predicting gaze data during reading with a gradient boosting decision tree approach,” in Proceedings of the NAACL Workshop on Cognitive Modeling and Computational Linguistics (Association for Computational Linguistics), 90–96. 10.18653/v1/2021.cmcl-1.10

[B7] Biedert R. Hees J. Dengel A. Buscher G. (2012). “A robust realtime reading-skimming classifier,” in Proceedings of the Symposium on Eye Tracking Research and Applications (New York, NY: Association for Computing Machinery), 123–130. 10.1145/2168556.2168575

[B8] Bonhage C. E. Mueller J. L. Friederici A. D. Fiebach C. J. (2015). Combined eye tracking and fMRI reveals neural basis of linguistic predictions during sentence comprehension. Cortex 68, 33–47. 10.1016/j.cortex.2015.04.011 26003489

[B9] Brainard D. H. (1997). The psychophysics toolbox. Spat. Vis. 10, 433–436. 10.1163/156856897X00357 9176952

[B10] Brennan J. (2016). Naturalistic sentence comprehension in the brain. Lang. Linguist. Compass 10, 299–313. 10.1111/lnc3.12198

[B11] Brennan J. R. Hale J. T. (2019). Hierarchical structure guides rapid linguistic predictions during naturalistic listening. PLoS ONE 14, e0207741. 10.1371/journal.pone.0207741 30650078 PMC6334990

[B12] Broderick M. P. Anderson A. J. Di Liberto G. M. Crosse M. J. Lalor E. C. (2018). Electrophysiological correlates of semantic dissimilarity reflect the comprehension of natural, narrative speech. Curr. Biol. 28, 803–809. 10.1016/j.cub.2018.01.080 29478856

[B13] Brouwer H. Crocker M. W. Venhuizen N. J. Hoeks J. C. (2017). A neurocomputational model of the N400 and the P600 in language processing. Cogn. Sci. 41, 1318–1352. 10.1111/cogs.12461 28000963 PMC5484319

[B14] Bruns A. (2004). Fourier-, hilbert-and wavelet-based signal analysis: are they really different approaches? J. Neurosci. Methods 137, 321–332. 10.1016/j.jneumeth.2004.03.002 15262077

[B15] Ceh S. M. Annerer-Walcher S. Koschutnig K. Körner C. Fink A. Benedek M.. (2021). Neurophysiological indicators of internal attention: an fMRI-eye-tracking coregistration study. Cortex 143, 29–46. 10.1016/j.cortex.2021.07.005 34371378

[B16] Chiang K.-J. Wei C.-S. Nakanishi M. Jung T.-P. (2019). “Cross-subject transfer learning improves the practicality of real-world applications of brain-computer interfaces,” in 9th International IEEE/EMBS Conference on Neural Engineering (San Francisco, CA), 424–427. 10.1109/NER.2019.8716958

[B17] Choi W. Desai R. H. Henderson J. M. (2014). The neural substrates of natural reading: a comparison of normal and nonword text using eyetracking and fmri. Front. Hum. Neurosci. 8, 1024. 10.3389/fnhum.2014.01024 25566039 PMC4274877

[B18] Cole M. J. Gwizdka J. Liu C. Bierig R. Belkin N. J. Zhang X.. (2011). Task and user effects on reading patterns in information search. Interact. Comput. 23, 346–362. 10.1016/j.intcom.2011.04.007

[B19] Cop U. Dirix N. Drieghe D. Duyck W. (2017). Presenting GECO: an eyetracking corpus of monolingual and bilingual sentence reading. Behav. Res. Methods 49, 602–615. 10.3758/s13428-016-0734-0 27193157

[B20] Culotta A. McCallum A. Betz J. (2006). “Integrating probabilistic extraction models and data mining to discover relations and patterns in text,” in Proceedings of the Human Language Technology Conference of the North American Chapter of the Association of Computational Linguistics (New York, NY), 296–303. 10.3115/1220835.1220873

[B21] [Dataset] Hollenstein N. Tröndle M. Plomecka M. Kiegeland S. Özyurt Y. Jäger L. A.. (2021c). Reading task classification using EEG and eye-tracking data. arXiv [Preprint]. arXiv: 2112.06310. Available online at: https://arxiv.org/pdf/2112.06310.pdf 10.3389/fpsyg.2022.1028824 PMC9878684 36710838

[B22] [Dataset] Jäger L. Kern T. Haller P. (2021). Potsdam Textbook Corpus (PoTeC): Eye Tracking Data from Experts and Non-experts Reading Scientific Texts. Available on OSF. 10.17605/OSF.IO/DN5HP

[B23] de Cheveignè A. (2020). Zapline: a simple and effective method to remove power line artifacts. Neuroimage 207, 116356. 10.1016/j.neuroimage.2019.116356 31786167

[B24] Degno F. Loberg O. Zang C. Zhang M. Donnelly N. Liversedge S. P.. (2019). Parafoveal previews and lexical frequency in natural reading: evidence from eye movements and fixation-related potentials. J. Exp. Psychol. Gen. 148, 453. 10.1037/xge0000494 30335444 PMC6388670

[B25] Dehghani M. Boghrati R. Man K. Hoover J. Gimbel S. I. Vaswani A.. (2017). Decoding the neural representation of story meanings across languages. Hum. Brain Mapp. 38, 6096–6106. 10.1002/hbm.23814 28940969 PMC6867091

[B26] Devlin J. Chang M.-W. Lee K. Toutanova K. (2019). “BERT: pre-training of deep bidirectional transformers for language understanding,” in Proceedings of the 2019 Conference of the North American Chapter of the Association for Computational Linguistics: Human Language Technologies (Minneapolis, MN: Association for Computational Linguistics), 4171–4186.

[B27] Dimigen O. Sommer W. Hohlfeld A. Jacobs A. M. Kliegl R. (2011). Coregistration of eye movements and EEG in natural reading: analyses and review. J. Exp. Psychol. Gen. 140, 552. 10.1037/a0023885 21744985

[B28] Ehinger B. V. Dimigen O. (2019). Unfold: an integrated toolbox for overlap correction, non-linear modeling, and regression-based EEG analysis. PeerJ 7, e7838. 10.7717/peerj.7838 31660265 PMC6815663

[B29] Finke A. Essig K. Marchioro G. Ritter H. (2016). Toward FRP-based brain-machine interfaces–single–trial classification of fixation-related potentials. PLoS ONE 11, e0146848. 10.1371/journal.pone.0146848 26812487 PMC4727887

[B30] Flesch R. (1948). A new readability yardstick. J. Appl. Psychol. 32, 221. 10.1037/h0057532 18867058

[B31] Frank S. L. Aumeistere A. (2022). An eye-tracking-with-EEG coregistration corpus of narrative sentences. PsyArXiv. 10.31234/osf.io/j5fgd

[B32] Frank S. L. Otten L. J. Galli G. Vigliocco G. (2013). “Word surprisal predicts n400 amplitude during reading,” in Proceedings of the 51st Annual Meeting of the Association for Computational Linguistics (Volume 2: Short Papers) (Sofia, Bulgaria: Association for Computational Linguistics), 878–883.

[B33] Frey A. Lemaire B. Vercueil L. Guèrin-Duguè A. (2018). An eye fixation-related potential study in two reading tasks: reading to memorize and reading to make a decision. Brain Topogr. 31, 640–660. 10.1007/s10548-018-0629-8 29450807

[B34] Hamilton L. S. Huth A. G. (2020). The revolution will not be controlled: natural stimuli in speech neuroscience. Lang. Cogn. Neurosci. 35, 573–582. 10.1080/23273798.2018.1499946 32656294 PMC7324135

[B35] Hasson U. Egidi G. (2015). “What are naturalistic comprehension paradigms teaching us about language?” in Cognitive Neuroscience of Natural Language Use, ed R. M. Willems (Cambridge: Cambridge University Press), 228–255. 10.1017/CBO9781107323667.011

[B36] Hollenstein N. Barrett M. Troendle M. Bigiolli F. Langer N. Zhang C.. (2019a). Advancing NLP with cognitive language processing signals. arXiv [Preprint]. 10.48550/arXiv.1904.0268230531985

[B37] Hollenstein N. Beinborn L. (2021). “Relative importance in sentence processing,” in Proceedings of the *59th Annual Meeting of the Association for Computational Linguistics and the 11th International Joint Conference on Natural Language Processing* (Association for Computational Linguistics), 141–150. 10.18653/v1/2021.acl-short.19

[B38] Hollenstein N. Chersoni E. Jacobs C. L. Oseki Y. Prévot L. Santus E.. (2021a). “CMCL 2021 shared task on eye-tracking prediction,” in Proceedings of the Workshop on Cognitive Modeling and Computational Linguistics, 72–78. 10.18653/v1/2021.cmcl-1.7

[B39] Hollenstein N. de la Torre A. Langer N. Zhang C. (2019b). “CogniVal: a framework for cognitive word embedding evaluation,” in Proceedings of the 23nd Conference on Computational Natural Language Learning (Hong Kong: Association for Computational Linguistics), 538–549. 10.18653/v1/K19-1050

[B40] Hollenstein N. Renggli C. Glaus B. Barrett M. Troendle M. Langer N.. (2021b). Decoding EEG brain activity for multi-modal natural language processing. Front. Hum. Neurosci. 15, 378. 10.3389/fnhum.2021.659410 34326723 PMC8314009

[B41] Hollenstein N. Rotsztejn J. Troendle M. Pedroni A. Zhang C. Langer N.. (2018). ZuCo, a simultaneous EEG and eye-tracking resource for natural sentence reading. Sci. Data 5, 180291. 10.1038/sdata.2018.291 30531985 PMC6289117

[B42] Hollenstein N. Troendle M. Zhang C. Langer N. (2020). “ZuCo 2.0: a dataset of physiological recordings during natural reading and annotation,” in Proceedings of The 12th Language Resources and Evaluation Conference (Marseille), 138–146.

[B43] Jäger L. A. Makowski S. Prasse P. Liehr S. Seidler M. Scheffer T.. (2020). “Deep eyedentification: biometric identification using micro-movements of the eye,ℍ in Machine Learning and Knowledge Discovery in Databases. Proceedings of the 2019 European Conference on Machine Learning and Principles and Practice of Knowledge Discovery in Databases (Würzburg, Germany), 299–314. 10.1007/978-3-030-46147-8_18

[B44] Kandylaki K. D. Bornkessel-Schlesewsky I. (2019). From story comprehension to the neurobiology of language. Lang. Cogn. Neurosci. 34, 405–410. 10.1080/23273798.2019.1584679

[B45] Kelton C. Wei Z. Ahn S. Balasubramanian A. Das S. R. Samaras D.. (2019). “Reading detection in real-time,” in Proceedings of the 11th ACM Symposium on Eye Tracking Research and Applications (Denver, CO: ACM Press), 1–5. 10.1145/3314111.3319916

[B46] Kliegl R. Dambacher M. Dimigen O. Jacobs A. M. Sommer W. (2012). Eye movements and brain electric potentials during reading. Psychol. Res. 76, 145–158. 10.1007/s00426-011-0376-x 21915693

[B47] Lemhöfer K. Broersma M. (2012). Introducing LexTALE: a quick and valid lexical test for advanced learners of English. Behav. Res. Methods 44, 325–343. 10.3758/s13428-011-0146-0 21898159 PMC3356522

[B48] Lobo J. L. Ser J. D. De Simone F. Presta R. Collina S. Moravek Z. (2016). “Cognitive workload classification using eye-tracking and EEG data,” in Proceedings of the International Conference on Human-Computer Interaction in Aerospace (New York, NY), 1–8. 10.1145/2950112.2964585

[B49] Lopopolo A. Frank S. L. Van den Bosch A. Nijhof A. Willems R. M. (2018). “The Narrative Brain Dataset (NBD), an fMRI dataset for the study of natural language processing in the brain,” in LREC 2018 Workshop on Linguistic and Neuro-Cognitive Resources (LiNCR) (Paris: LREC), 8–11.

[B50] Luke S. G. Christianson K. (2018). The provo corpus: a large eye-tracking corpus with predictability norms. Behav. Res. Methods 50, 826–833. 10.3758/s13428-017-0908-4 28523601

[B51] Mathias S. Kanojia D. Mishra A. Bhattacharya P. (2020). “A survey on using gaze behaviour for natural language processing,” in Proceedings of the 29th International Joint Conference on Artificial Intelligence (Yohokama), 4907–4913. 10.24963/ijcai.2020/683

[B52] Mathur P. Mittal T. Manocha D. (2021). “Dynamic graph modeling of simultaneous EEG and eye-tracking data for reading task identification,” in IEEE International Conference on Acoustics, Speech and Signal Processing (Toronto, ON), 1250–1254. 10.1109/ICASSP39728.2021.9414343

[B53] MathWorks Inc. (2000). MATLAB: The Language of Technical Computing. External interfaces. MathWorks, Incorporated.

[B54] McGuire E. Tomuro N. (2021). “Relation classification with cognitive attention supervision,” in Proceedings of the Workshop on Cognitive Modeling and Computational Linguistics, 222–232. 10.18653/v1/2021.cmcl-1.26

[B55] Michel C. M. Koenig T. (2018). EEG microstates as a tool for studying the temporal dynamics of whole-brain neuronal networks: a review. Neuroimage 180, 577–593. 10.1016/j.neuroimage.2017.11.062 29196270

[B56] Miller B. W. (2015). Using reading times and eye-movements to measure cognitive engagement. Educ. Psychol. 50, 31–42. 10.1080/00461520.2015.1004068

[B57] Nastase S. A. Gazzola V. Hasson U. Keysers C. (2019). Measuring shared responses across subjects using intersubject correlation. Soc. Cogn. Affect. Neurosci. 14, 667–685. 10.1093/scan/nsz037 31099394 PMC6688448

[B58] Nastase S. A. Goldstein A. Hasson U. (2020). Keep it real: rethinking the primacy of experimental control in cognitive neuroscience. Neuroimage 222, 117254. 10.1016/j.neuroimage.2020.117254 32800992 PMC7789034

[B59] Nastase S. A. Liu Y.-F. Hillman H. Zadbood A. Hasenfratz L. Keshavarzian N.. (2021). Narratives: fMRI data for evaluating models of naturalistic language comprehension. bioRxiv. 10.1101/2020.12.23.424091 34584100 PMC8479122

[B60] Notaro G. M. Diamond S. G. (2018). Simultaneous EEG, eye-tracking, behavioral, and screen-capture data during online German language learning. Data Brief 21, 1937–1943. 10.1016/j.dib.2018.11.044 30519619 PMC6260224

[B61] Pedroni A. Bahreini A. Langer N. (2019). Automagic: standardized preprocessing of big EEG data. Neuroimage 200, 460–473. 10.1016/j.neuroimage.2019.06.046 31233907

[B62] Pereira F. Lou B. Pritchett B. Ritter S. Gershman S. J. Kanwisher N.. (2018). Toward a universal decoder of linguistic meaning from brain activation. Nat. Commun. 9, 963. 10.1038/s41467-018-03068-4 29511192 PMC5840373

[B63] Pfeiffer C. Hollenstein N. Zhang C. Langer N. (2020). Neural dynamics of sentiment processing during naturalistic sentence reading. Neuroimage 218, 116934. 10.1016/j.neuroimage.2020.116934 32416227

[B64] Pion-Tonachini L. Kreutz-Delgado K. Makeig S. (2019). Iclabel: an automated electroencephalographic independent component classifier, dataset, and website. Neuroimage 198, 181–197. 10.1016/j.neuroimage.2019.05.026 31103785 PMC6592775

[B65] Raatikainen P. Hautala J. Loberg O. Kärkkäinen T. Leppänen P. Nieminen P.. (2021). Detection of developmental dyslexia with machine learning using eye movement data. Array 12, 100087. 10.1016/j.array.2021.100087

[B66] Rämä P. Baccino T. (2010). Eye fixation-related potentials (EFRPs) during object identification. Vis. Neurosci. 27, 187–192. 10.1017/S0952523810000283 20939937

[B67] Rello L. Ballesteros M. (2015). “Detecting readers with dyslexia using machine learning with eye tracking measures,” in Proceedings of the 12th International Web for All Conference (New York, NY: Association for Computing Machinery), 1–8. 10.1145/2745555.2746644

[B68] Schalk G. Brunner P. Gerhardt L. A. Bischof H. Wolpaw J. R. (2008). Brain-computer interfaces (BCIS): detection instead of classification. J. Neurosci. Methods 167, 51–62. 10.1016/j.jneumeth.2007.08.010 17920134

[B69] Scheinost D. Noble S. Horien C. Greene A. S. Lake E. M. R. Salehi M.. (2019). Ten simple rules for predictive modeling of individual differences in neuroimaging. Neuroimage 193, 35–45. 10.1016/j.neuroimage.2019.02.057 30831310 PMC6521850

[B70] Sereno S. C. Rayner K. (2003). Measuring word recognition in reading: eye movements and event-related potentials. Trends Cogn. Sci. 7, 489–493. 10.1016/j.tics.2003.09.010 14585445

[B71] Shain C. Blank I. A. van Schijndel M. Schuler W. Fedorenko E. (2020). fMRI reveals language-specific predictive coding during naturalistic sentence comprehension. Neuropsychologia 138, 107307. 10.1016/j.neuropsychologia.2019.107307 31874149 PMC7140726

[B72] Sun P. Anumanchipalli G. K. Chang E. F. (2020). Brain2Char: a deep architecture for decoding text from brain recordings. J. Neural Eng. 17, 066015. 10.1088/1741-2552/abc742 33142282 PMC9591243

[B73] Tokunaga T. Nishikawa H. Iwakura T. (2017). An eye-tracking study of named entity annotation. Proceedings of the International Conference Recent Advances in Natural Language Processing (Varna, Bulgaria: INCOMA Ltd.), 758–764. 10.26615/978-954-452-049-6_097

[B74] Tomanek K. Hahn U. Lohmann S. Ziegler J. (2010). “A cognitive cost model of annotations based on eye-tracking data,” in Proceedings of the 48th Annual Meeting of the Association for Computational Linguistics (Uppsala, Sweden), 1158–1167.

[B75] Tor H. T. Ooi C. P. Lim-Ashworth N. S. J. Wei J. K. E. Jahmunah V. Oh S. L.. (2021). Automated detection of conduct disorder and attention deficit hyperactivity disorder using decomposition and nonlinear techniques with EEG signals. Comput. Methods Programs Biomed. 200, 105941. 10.1016/j.cmpb.2021.105941 33486340

[B76] Wang Z. Ji H. (2021). Open vocabulary electroencephalography-to-text decoding and zero-shot sentiment classification. arXiv. [preprint]. 10.48550/arXiv.2112.02690

[B77] Yadav D. Jain R. Agrawal H. Chattopadhyay P. Singh T. Jain A.. (2019). EvalAI: towards better evaluation systems for AI agents. arXiv. [preprint]. 10.48550/arXiv.1902.03570

