# Supplementary material for: The ZuCo benchmark on cross-subject reading task classification with EEG and eye-tracking data
Source: Front Psychol. 2023 Jan 12;13:1028824. doi: 10.3389/fpsyg.2022.1028824 (PMC9878684; doi:10.3389/fpsyg.2022.1028824)
Supplement: Supplementary file 1 [file Data_Sheet_1.pdf]

1 APPENDIX

Features	XBB	XDT	XLS	XPB	XSE	XTR	XWS	XAH	XBD	XSS
sent_gaze_sacc	0.66	0.47	0.69	0.62	0.81	0.66	0.78	0.7	0.79	0.68
sent_gaze_sacc_eeg_means	0.64	0.48	0.69	0.64	0.78	0.57	0.81	0.74	0.74	0.69
electrode_features_all	0.58	0.45	0.53	0.56	0.57	0.49	0.64	0.5	0.57	0.64
electrode_features_all_pca	0.47	0.58	0.52	0.57	0.61	0.68	0.66	0.62	0.53	0.54

Table 8. Mean accuracy after bootstrapping the testset using 500 samples and a sample size equal to the original data for each subject in the held-out testset and each feature-set.

Features	XBB	XDT	XLS	XPB	XSE	XTR	XWS	XAH	XBD	XSS
sent_gaze_sacc	0.65	0.4	0.67	0.58	0.81	0.66	0.78	0.68	0.79	0.66
sent_gaze_sacc_eeg_means	0.63	0.44	0.67	0.6	0.76	0.51	0.81	0.74	0.73	0.67
electrode_features_all	0.37	0.32	0.42	0.46	0.57	0.47	0.63	0.38	0.37	0.6
electrode_features_all_pca	0.44	0.57	0.51	0.56	0.58	0.67	0.65	0.61	0.5	0.52

Table 9. Mean f1-score after bootstrapping the testset using 500 samples and a sample size equal to the original data for each subject in the held-out testset and each feature-set.

Features	XBB	XDT	XLS	XPB	XSE	XTR	XWS	XAH	XBD	XSS
sent_gaze_sacc	0.7	0.48	0.7	0.74	0.81	0.69	0.82	0.71	0.83	0.71
sent_gaze_sacc_eeg_means	0.63	0.5	0.72	0.75	0.81	0.72	0.83	0.74	0.81	0.74
electrode_features_all	0.29	0.31	0.66	0.77	0.58	0.47	0.68	0.37	0.49	0.65
electrode_features_all_pca	0.44	0.57	0.52	0.58	0.59	0.66	0.68	0.61	0.5	0.52

Table 10. Mean precision after bootstrapping the testset using 500 samples and a sample size equal to the original data for each subject in the held-out testset and each feature-set.

Features	XBB	XDT	XLS	XPB	XSE	XTR	XWS	XAH	XBD	XSS
sent_gaze_sacc	0.68	0.49	0.68	0.63	0.8	0.67	0.79	0.69	0.8	0.67
sent_gaze_sacc_eeg_means	0.63	0.5	0.68	0.65	0.75	0.59	0.82	0.74	0.75	0.68
electrode_features_all	0.5	0.46	0.54	0.56	0.59	0.48	0.65	0.44	0.5	0.61
electrode_features_all_pca	0.45	0.57	0.52	0.57	0.58	0.65	0.66	0.61	0.5	0.52

Table 11. Mean recall after bootstrapping the testset using 500 samples and a sample size equal to the original data for each subject in the held-out testset and each feature-set.

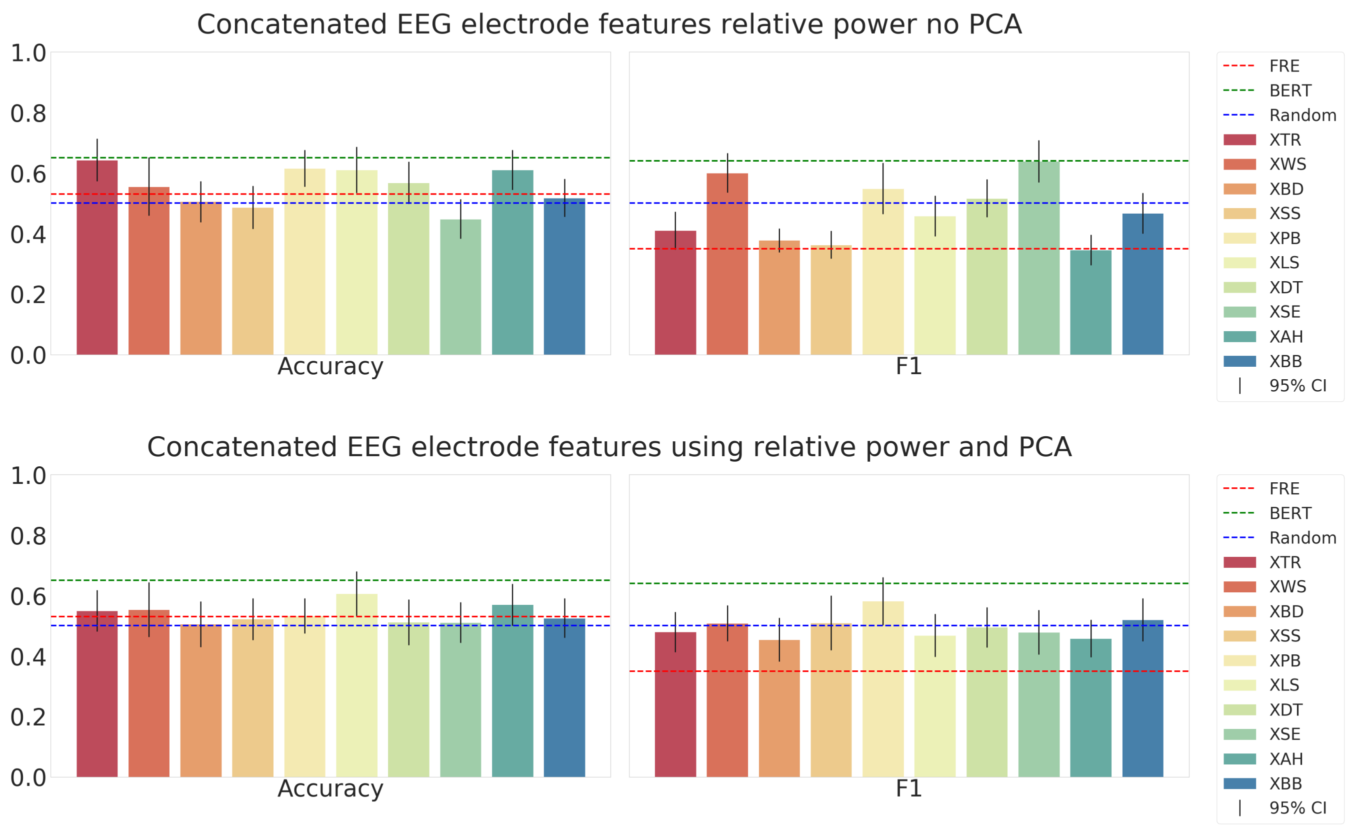

Figure 13. The mean accuracy and mean F1-score with 95% confidence intervals and textual baselines are plotted for each subject in the held-out test dataset using the concatenated EEG electrode features with relative EEG power.
